# Defect Density Analysis of WO_***x***_ and MoO_***x***_ Thin Films Grown by Pulsed Laser Deposition for Heterojunction Solar Cell Applications

**DOI:** 10.1021/acsaem.5c00629

**Published:** 2025-06-23

**Authors:** Daniele Scirè, Roberto Macaluso, Mauro Mosca, Maria Pia Casaletto, Olindo Isabella, Miro Zeman, Isodiana Crupi

**Affiliations:** † Department of Engineering, 18998 University of Palermo, Viale delle Scienze, Ed. 9, Palermo 90128, Italy; ‡ Institute of Nanostructured Materials (ISMN), 228703 National Research Council (CNR), Via Ugo La Malfa 153, Palermo 90146, Italy; § Photovoltaic Materials and Devices Group, 2860 Delft University of Technology, Mekelweg 4, Delft 2628CD, Netherlands

**Keywords:** transition metal oxides, molybdenum oxide, tungsten oxide, heterojunction solar cells, defect density, photothermal deflection spectroscopy, pulsed laser deposition

## Abstract

This study presents a comprehensive analysis of the optical and electronic properties of thin films of molybdenum oxide and tungsten oxide to implement hole-selective contact for heterojunction solar cells. These contacts are currently viewed as an alternative for the fabrication of doping-free solar cells. However, the spreading of this technology is still limited due to the development of S-shaped *J*–*V* curves, which affect the electrical performance of the cells, and further optimization in the material deposition process is therefore crucial to overcome these challenges. To improve transition metal oxide-based heterojunction technology, this work investigates the impact of oxygen vacancies on electrical performance, particularly their role in S-shaped *J*–*V* curves. Defect density evaluation through nondestructive techniques like photothermal deflection spectroscopy together with a detailed experimental characterization is presented in this paper to highlight the structural and optical properties of the films. Prototypes of solar cells incorporating hole-selective contacts with tungsten and molybdenum oxide are prepared to show S-shaped *J*–*V* characteristics under AM 1.5 illumination. An equivalent circuit modeling was used for understanding the electrical characteristics of the prototypes. Furthermore, this approach offers insights into the optimization of the performances of devices.

## Graphical abstract

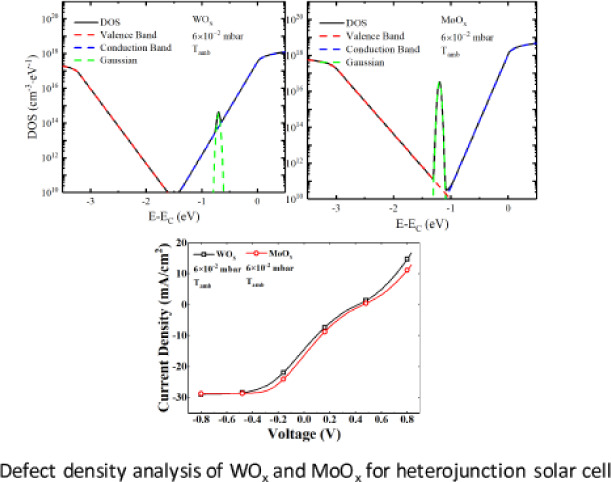

## 1 Introduction

The increasing demand for reliable and cost-effective renewable energy sources has encouraged interest in solar energy adoption, with the photovoltaic (PV) industry relying predominantly on silicon-based technology with crystalline-silicon (c-Si) technology alone accounting for 97% of the production.^1,2^ In recent years, the development of c-Si heterojunction technology (HJT) has enabled the production of solar cells with record efficiencies of 27.3% obtained by LONGi in 2024.^3,4^ The HJT technology is based on the implementation of passivation layers of hydrogenated-amorphous silicon (a-Si:H); however, its utilization introduces challenges due to defects and parasitic absorption, which represent obstacles in the optimization of the solar cell.^5^

In response to these challenges, researchers are exploring alternative materials, such as TMOs (transition metal oxides) in the substitution of a-Si:H in HJT and implementation of passivating carrier-selective contacts (CSC). Among these, molybdenum trioxide and tungsten trioxide for hole-selective contacts^6–10^ and titanium dioxide for electron-selective contacts^11,12^ are being investigated for their potential. Transition metal oxides have also gained significant attention in applied energy materials due to their versatile electronic properties and catalytic activity. Beyond their role in photovoltaic applications, TMOs are widely explored as efficient photocatalysts and electrocatalysts.^13,14^ These materials can be deposited by using various techniques, such as atomic layer deposition (ALD), sputtering, and pulsed laser deposition (PLD). Initial studies employing TMO-based selective contacts have demonstrated promising efficiencies,^15–20^ but further optimization is necessary. Moreover, the development of TMO-based solar cells faces several challenges, particularly in addressing the development of S-shaped *J*–*V* curves,^21–23^ which significantly lower electrical performance by reducing the fill factor and increasing resistivity losses.

In this paper, substoichiometric molybdenum oxide MoO_*x*_ and tungsten oxide WO_*x*_ (*x* ≈3) deposited via PLD are investigated as hole-selective contacts. To achieve efficient hole-selective contacts, it is crucial to optimize the charge transport properties of transition metal oxides, which depend on oxygen stoichiometry, the work function, and defect states. Pulsed laser deposition allows precise control over these parameters, making it a valuable tool for studying the fundamental properties. Although PLD is not typically used for large-scale manufacturing, the insights gained can guide the development of scalable deposition techniques, such as sputtering or atomic layer deposition. Furthermore, it offers excellent control over film stoichiometry and structure, making it ideal for fundamental investigations into defect formation and electronic behavior. This work is based on PLD to explore the relationship between materials, their properties, and performances that can be used as a precursor to other scalable deposition approaches.

Understanding the role of bandgap defects of these materials, particularly oxygen vacancies, is essential for addressing S-shaped *J*–*V* curves and advancing TMO-based heterojunction technology. This study systematically explores how deposition conditions influence defect formation and carrier selectivity to provide a foundation for process optimization. Defect density evaluation through techniques such as photothermal deflection spectroscopy (PDS) provides valuable insights for optimization. Detailed experimental characterization, including X-ray photoelectron spectroscopy (XPS), Raman spectroscopy, and optical measurements, reveals the structural and optical properties of the TMO films. This comprehensive analysis aids in understanding material behavior to optimize fabrication processes. Prototypes of HJT solar cells incorporating TMO-based hole-selective contacts exhibit S-shaped *J*–*V* characteristics under an AM 1.5 illumination. To address this behavior, an equivalent circuit modeling, incorporating a series Schottky junction, provides a framework for understanding and analyzing the electrical characteristics of the solar cells under study and giving insights into the optimization of the device’s performance. S-shaped *J*–*V* characteristics in TMO-based heterojunctions are commonly associated with charge extraction barriers, poor band alignment, or interface recombination.^12,23,24^ Understanding their origin is critical to unlocking the full potential of TMOs in silicon photovoltaics, yet their correlation with optoelectronic properties remains insufficiently explored. Despite the promising application of transition metal oxides as hole-selective contacts, the fundamental understanding of how oxygen-related defects impact their electronic properties and device performance remains limited. This study aims to correlate subgap absorption, linked to oxygen vacancies, with key photovoltaic parameters in MoO_*x*_ and WO_*x*_ films deposited via PLD.

## 2 Experimental Details

WO_*x*_ and MoO_*x*_ thin films were deposited onto commercially available fused silica substrates by PLD. The PLD system employs a frequency-tripled Nd:YAG laser from Quantel mod. YG78C20 with λ = 355 nm.^25–27^ As precursors, tablets (diameter of 1.5 in.) of stoichiometric tungsten trioxide (WO_3_, 99.9%) and molybdenum trioxide (MoO_3_, 99.9%) were used as targets. Before each deposition, substrates were cleaned in an ultrasonic bath with acetone, rinsed with isopropanol, and dried with compressed air. Each deposition was carried out by varying the deposition condition in terms of the substrate temperature, *T*_dep_, and deposition chamber oxygen pressure, PO_2_. For each material, six samples were fabricated with PO_2_ = 3 × 10^–2^, 6 × 10^2^, and 10 × 10^2^ mbar at room temperature and at 200 °C. For comparison purposes, an additional sample was produced for each material using a PO_2_ = 3 × 10^–2^ mbar and a deposition temperature of 400 °C to induce crystallization of the films. The laser energy density (fluency) was maintained at 1.2 J/cm^2^, and the repetition rate was 20 Hz. A summary of the parameters regarding the PLD and the depositions is reported in Table S1. Finally, an *x*–*y* microtranslator system was employed to ensure the target surface’s uniform ablation.

XPS measurements were carried out by a VG Microtech ESCA3000 Multilab spectrometer, equipped with a twin (Mg and Al) anode and a five-channeltron detection system. An Al Kα (*h*ν = 1486.6 eV) X-ray source and a hemispherical analyzer (CAE mode) operated in an ultrahigh vacuum chamber (base pressure less than 1 × 10^–6^ Pa). Photoelectron signals and peak components were assigned according to XPS reference database.^28^ The binding energy (BE) scale was calibrated by measuring the C 1s peak at a BE of 285.1 eV from surface contamination. The accuracy of the BE measure was ±0.1 eV. Photoemission data were collected and processed by using VGX900 and XPSPeak software, respectively.

The structural evaluation of the oxide films has been assessed by Raman spectroscopy obtained through a Renishaw inVia Micro-Raman microscope working at a wavelength of 514 nm.

A photothermal deflection spectroscopy setup, described elsewhere,^29^ was used to measure the absorption coefficient of the samples. The PDS directly measures the absorption in thin film deposited on a substrate (fused silica or quartz) by measuring the deflection of a He–Ne laser beam tangent to the sample edge submerged in a cuvette filled with a liquid (FC-72). This deflection is proportional to the absorption within the sample and exhibits superior sensitivity over transmittance/reflectance spectrophotometry; as such, it has been extensively used to analyze electronic defects in semiconductors and amorphous transition metal oxides.^8,12,15^

The solar cell prototypes were fabricated following the process described elsewhere:^17^ initially, the electron-selective contact was formed on top of a c-Si­(n) wafer by plasma-enhanced chemical vapor deposition (PECVD) with 8 nm of a-Si:H­(i) passivation layer, followed by 11 nm of nc-SiO_*x*_:H­(n)/nc-Si:H­(n); afterward, a passivation layer of 9 nm of a-Si:H­(i) was deposited by PECVD on the backside. Subsequently, the wafer was diced into 1.5 × 1.5 cm^2^ squares for the deposition of the hole-selective contact. The cells were divided into two sets of three cells each: MoO_*x*_ was used in one set while WO_*x*_ was used for the other one. These layers of TMO were deposited on top of the 9 nm a-Si:H­(i) passivation layer at ambient temperature, to avoid crystallization of the amorphous silicon, and three different oxygen pressures of 3 × 10^–2^, 6 × 10^2^, and 10 × 10^2^ mbar. As a final step, the cells were completed by sputtering 70 nm of indium tin oxide (ITO) as an antireflection coating on the front side and by thermal evaporation of 1 μm-thick aluminum on both faces for metallization. The bottom contact was obtained with a planar Al deposition onto the nc-SiO_*x*_:H/nc-Si:H film, while the top contact, with a grid pattern, was deposited onto the ITO using a shadow mask. A graphical representation of the solar cell stack is depicted in Figure 1.

**Figure 1:**
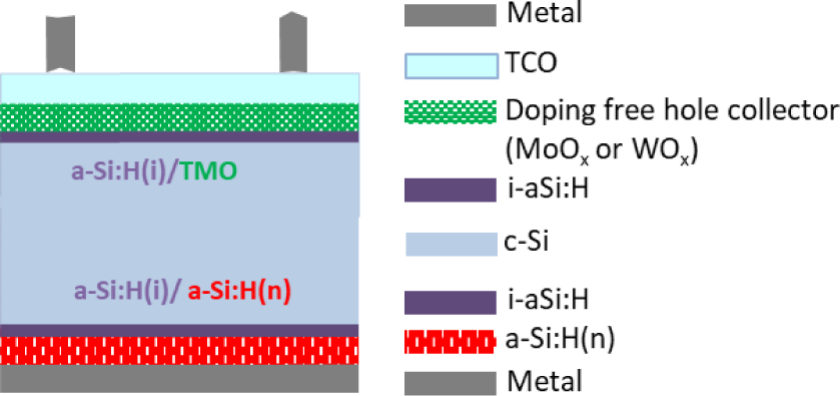
Graphical representation of the solar cell prototypes realized by implementing a hole selective contact made of MoO_*x*_ or WO_*x*_. The structure of the cell is composed of a stack of c-Si passivated with aSi:H, the electron selective contact is made of a-Si:H­(n), and the hole-selective contact is made, alternatively, of MoO_*x*_ or WO_*x*_.

## 3 Results

### 3.1 Surface Chemical Composition

XPS survey spectra of WO_*x*_ samples deposited at ambient temperature with PO_2_ = 3 × 10^–2^ mbar and PO_2_ = 10 × 10^–2^ mbar are shown in Figure 2a,b, respectively. The presence of tungsten peaks (W 4f and W 4d) and oxygen (O 1s) is detected, along with carbon (C 1s) due to surface impurity.

**Figure 2:**
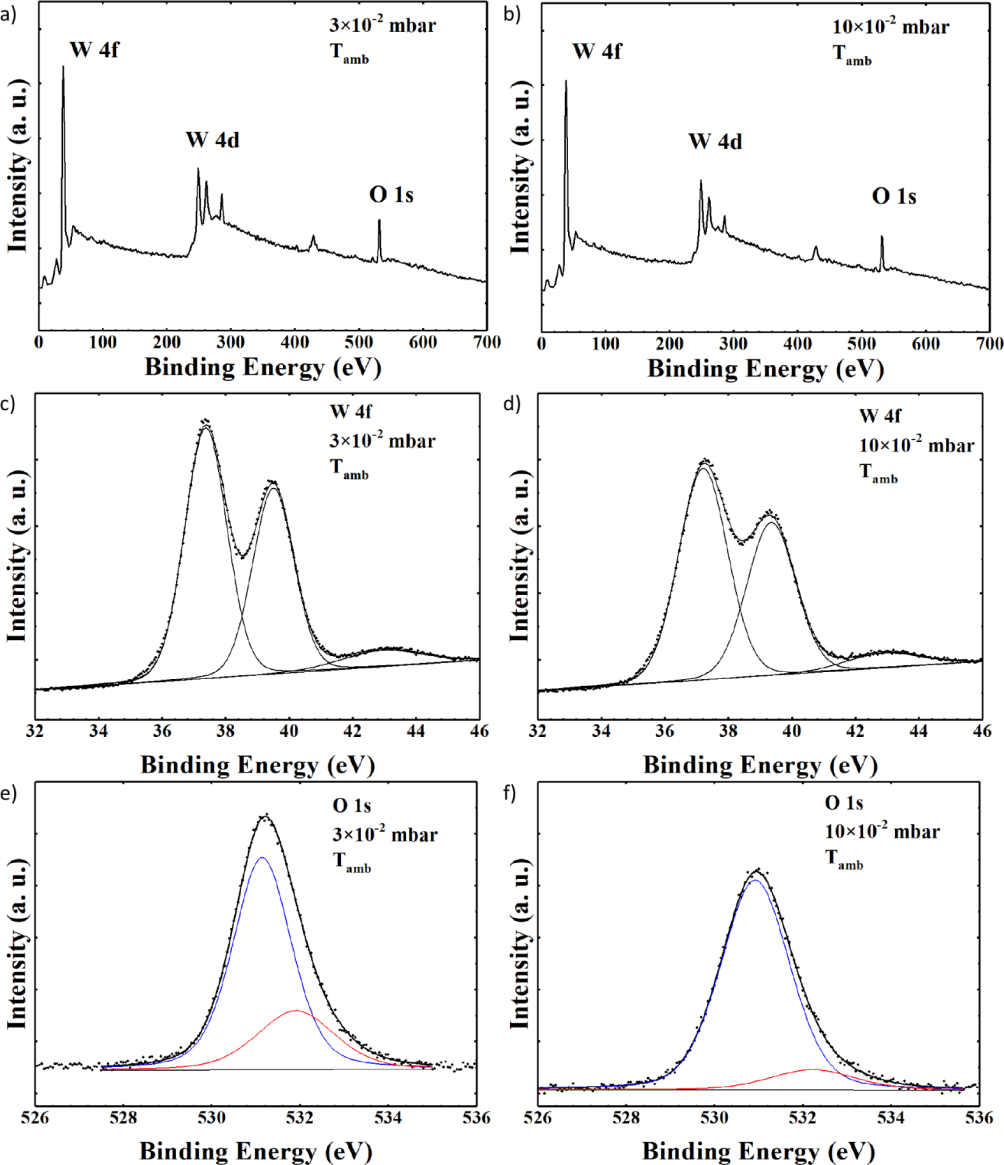
XPS spectra for WO_*x*_ samples deposited at *T*_amb_ with PO_2_ = 3 × 10^–2^ mbar: (a) wide scan, (c) curve fitting of the W 4f spectrum, (e) curve fitting of the O 1s spectrum; and with PO_2_ = 10 × 10^–2^ mbar: (b) wide scan, (d) curve fitting of the W 4f spectrum, and (f) curve fitting of the O 1s spectrum.

The curve-fitting of W 4f spectra is shown in Figure 2c,d. In both samples, the W 4f_7/2_ component of the (W 4f_7/2_ – W 4f_5/2_) doublet is located at BE = 37.4 eV and assigned to WO_3_ oxide.^28^ The curve-fitting of O 1s spectra reported in Figure 2e,f for both samples resulted in two components, a major one located at BE = 531.2 eV and assigned to WO_3_ oxide and a minor one located at BE = 532.4 eV attributed to the substoichiometric WO_*x*_ oxide.^28^ Both samples are substoichiometric, showing a very similar surface chemical composition, with no substantial differences. Results of the XPS analysis are summarized in Table 1. For comparison, results of the surface XPS analysis of the WO_*x*_ sample deposited at 400 °C with a pressure *PO*_2_ = 3 × 10^–2^ mbar are shown in Figures S1–S3 and listed in Table S2.

**Table 1:** XPS Curve Fitting of Tungsten Oxide and Molybdenum Oxide Samples^a^

	WO_*x*_				MoO_*x*_			
Sample	BE (eV)		Concentration (at. %)		BE (eV)		Concentration (at. %)	
PO_2_, *T*_dep_	W 4f_7/2_	W 4f_5/2_	W	O	Mo 3d_5/2_	Mo 3d_3/2_	Mo	O
	O 1s		O/W		O 1s		O/Mo	
10 × 10^–2^ mbar, *T*_amb_	37.2	39.3	55.2	44.8	233.4	236.6	48.9	51.1
	530.9		0.8		530.9		1.1	
	532.2				532.5			
3 × 10^–2^ mbar, *T*_amb_	37.3	39.5	55.3	44.7	233.4	236.5	49.5	50.5
	531.1		0.8		530.9		1.0	
	531.9				532.4			

a The two spin-orbit components (W 4f_7/2_ and W 4f_5/2_) of the W 4f spectrum resulted in energy split by Δ = 2.14 eV. The two spin-orbit components (mo 3d_5/2_ and Mo 3d_3/2_) of the Mo 3d spectrum were energy split by Δ ∼3.15 eV. Elemental concentration is expressed as atomic percentage (at. %).

XPS survey spectra of MoO_*x*_ samples deposited at ambient temperature with PO_2_ = 3 × 10^–2^ mbar and PO_2_ = 10 × 10^–2^ mbar are shown in Figure 3a,b, respectively. The photoelectron signals of molybdenum (Mo 3p and Mo 3d) and oxygen (O 1s) are detected, along with C 1s due to surface impurity. The curve fitting of Mo 3d spectra is shown in Figure 3c,d. In both samples, the Mo 3d_5/2_ component of the (Mo 3d_5/2_ – Mo 3d_3/2_) doublet peak is located at BE = 233.4 eV and assigned to MoO_3_ oxide.^28^ The curve fitting of the O 1s spectra for both samples deposited at room temperature is reported in Figure 3e,f. Two peak components were found: a major one located at BE = 530.9 eV and assigned to MoO_3_ oxide and a minor one located at BE = 532.5 eV and assigned to the substoichiometric MoO_*x*_ oxide.^28^ The presence of substoichiometric oxide is confirmed for all the samples. Results of the XPS analysis are summarized in Table 1. For comparison, results of the XPS analysis of the sample deposited at 400 °C with a pressure PO_2_ = 3 × 10^–2^ mbar are shown in Figures S4–S6 and summarized in Table S3.

**Figure 3:**
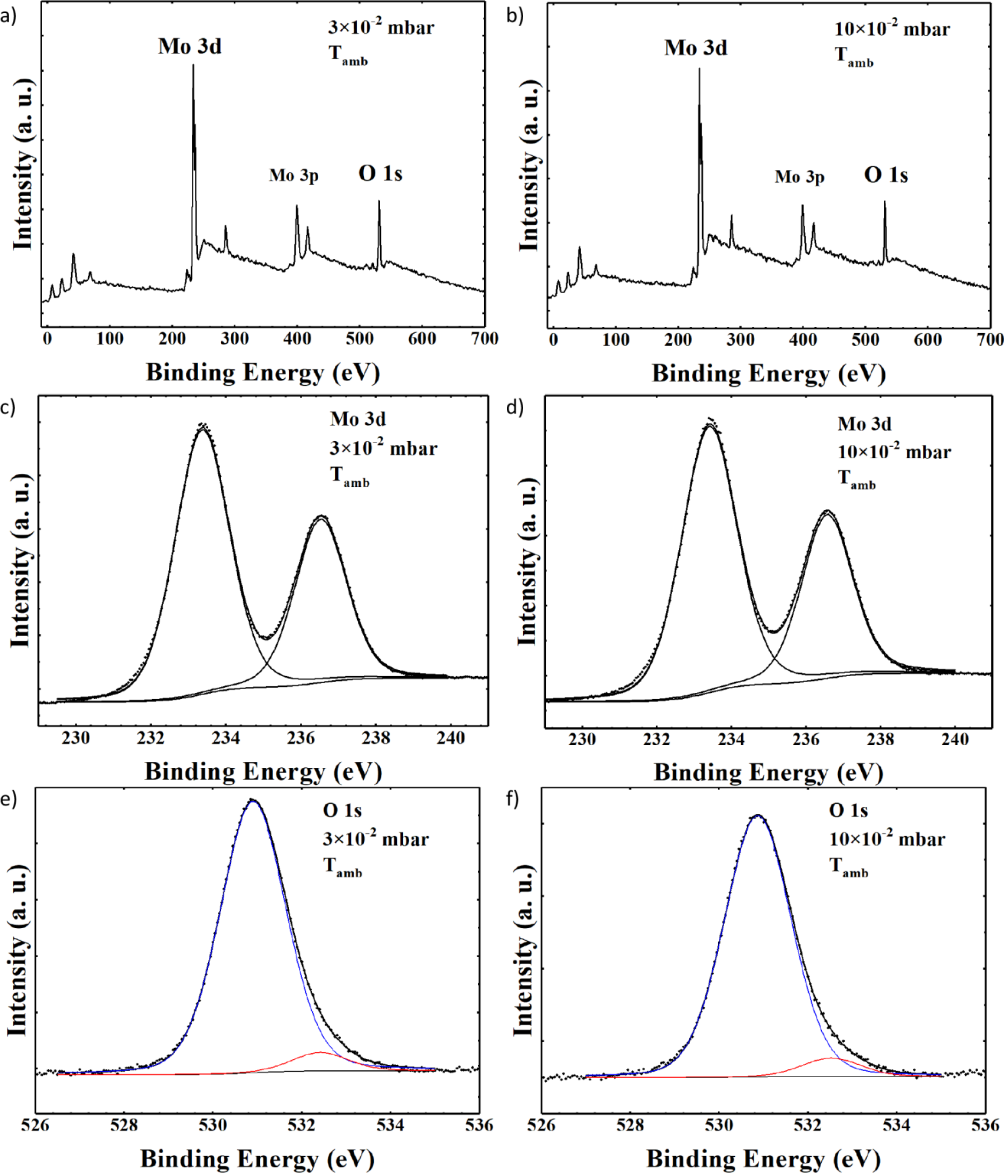
XPS spectra for MoO_*x*_ samples deposited at *T*_amb_ with PO_2_ = 3 × 10^–2^ mbar: (a) wide scan, (c) curve fitting of the Mo 3d spectrum, (e) curve fitting of the O 1s spectrum; and with PO_2_ = 10 × 10^–2^ mbar: (b) wide scan, (d) curve fitting of the Mo 3d spectrum, and (f) curve fitting of the O 1s spectrum.

Previous studies report that the work function of MoO_*x*_ and WO_*x*_ is strongly influenced by oxygen stoichiometry, typically ranging from 5.4 to 6.9 eV for MoO_*x*_ and from 5.1 to 6.8 eV for WO_*x*_.^30–37^ The XPS analysis confirms a variation in oxidation states with an increase in oxygen pressure, suggesting an improvement in work function alignment with the silicon substrate. This is crucial for hole transport, as a higher work function reduces the energy barrier at the TMO/c-Si interface, thus enhancing charge extraction and mitigating resistive losses.

### 3.2 Structural Characterization

Figure 4a shows the Raman spectra of the tungsten oxide samples. The absence of any peaks deposited at ambient temperature and 200 °C highlights the fact that these samples are amorphous. Instead, Figure 4b depicts the Raman spectrum of the sample deposited at 400 °C, which shows several sharp peaks at 273 cm^–1^, 712 cm^–1^, and 805 cm^–1^, highlighting a crystalline phase with a structure made of clusters of WO_4_ tetrahedral cells and WO_6_ octahedral cells.^38^ The peak at 273 cm^–1^ represents the vibrations δ­(O–W–O), and those at 712 cm^–1^ and 805 cm^–1^ represent the vibrational modes of the O–W–O bonds. In the spectrum, there is also a small peak around 950 cm^–1^ attributable to the W=O bonds.^39^

**Figure 4:**
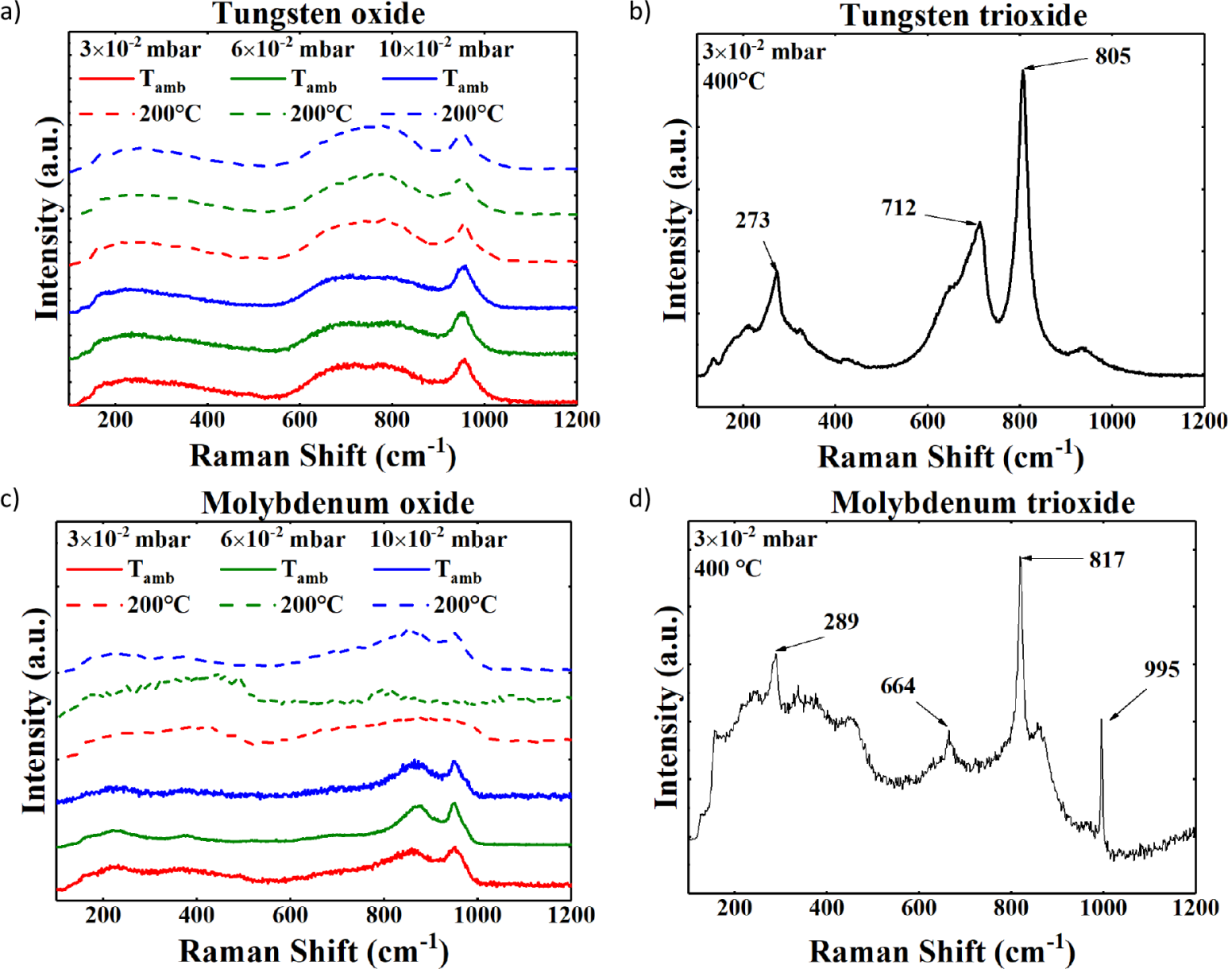
Raman spectra of the substoichiometric tungsten oxide samples deposited at (a) ambient temperature and 200 °C compared with (c) the tungsten trioxide deposited at 400 °C. Raman spectra of the substoichiometric molybdenum oxide samples deposited at (b) ambient temperature and 200 °C compared with (d) the molybdenum trioxide deposited at 400 °C.

Figure 4c shows the Raman spectra of the molybdenum oxide samples. The sample deposited at ambient temperature shows two small and broad peaks at 879 cm^–1^ and 952 cm^–1^, which are not indicative of a crystalline phase but most likely a local cluster of polycrystalline substoichiometric MoO_*x*_.^40,41^ Furthermore, the samples deposited at 200 °C show no peaks, except for one deposited with the highest oxygen pressure, which exhibits a similar spectrum to those deposited at ambient temperature. Besides, the spectra shown in Figure 4c cannot be associated with any crystalline phase, concluding that all these films are amorphous. Finally, the Raman spectrum of a crystalline sample is shown in Figure 4d where the deposition temperature is raised to 400 °C. Such a spectrum shows several sharp peaks at 289 cm^–1^, 664 cm^–1^, 817 cm^–1^, and 995 cm^–1^ referred to the Mo=O bonds^40,41^ of the monocrystalline orthorhombic α-MoO_3_ phase.^40^

### 3.3 Optical Characterization

Figure 5a shows the absorption coefficients of the substoichiometric WO_*x*_ samples. The data are color-coded according to the oxygen partial pressure used during the deposition. Furthermore, solid lines correspond to ambient temperature depositions, while dashed lines represent films deposited at 200 °C. Generally, it can be noted that all spectra progressively increase from lower energies, and beyond 3 eV, they rise exponentially to a final plateau in the UV region for an energy of above 3.8 eV. Notably, from 3 to 4 eV, the absorption spectra of the films deposited at ambient temperature are lower than those deposited under the same PO_2_ conditions at 200 °C. The sample deposited with an oxygen pressure of 10 × 10^–2^ mbar at 200 °C exhibits the highest absorption for a broad energy range, whereas the lowest absorption is achieved with the sample deposited at room temperature and 10 × 10^–2^ mbar oxygen pressure.

**Figure 5:**
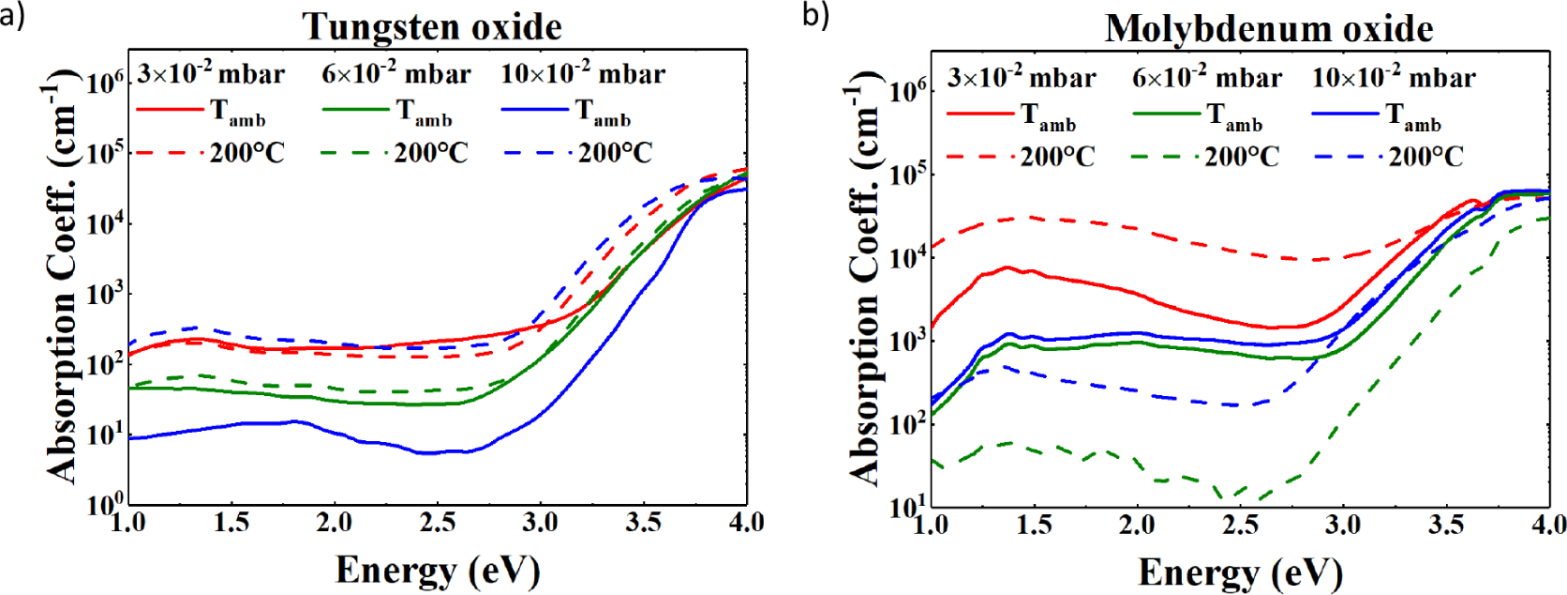
Absorption coefficient of the substoichiometric (a) WO_*x*_ and (b) MoO_*x*_ samples grown under different deposition conditions.

For the tungsten oxide samples, the observed absorption increase in the subgap region (below 3 eV) with higher deposition temperature and intermediate PO_2_ values suggests a greater density of defect-related states, likely due to oxygen vacancies or structural disorder. The enhanced absorption below the optical bandgap is consistent with the presence of localized states in the bandgap, which facilitate transitions observable in PDS. These trends indicate that thermal energy during deposition plays a critical role in enhancing the defectivity of the deposited WO_*x*_ samples, contributing to subgap absorption and therefore highlighting a peak in the absorption spectrum.

Figure 5b shows the absorption coefficients of the substoichiometric MoO_*x*_ samples. The experimental data are grouped by a color for a specific oxygen deposition pressure, whereas the full line groups the data with the same deposition temperature (ambient temperature, full line; 200 °C, dashed line). The film deposited with PO_2_ = 3 × 10^–2^ mbar at 200 °C shows the highest absorption for nearly all energy ranges. Notably, the samples deposited at PO_2_ = 6 × 10^–2^ mbar and PO_2_ = 10 × 10^–2^ mbar at ambient temperature exhibit a comparable spectrum.

The absorption behavior of the MoO_*x*_ samples reveals a nonmonotonic dependence on the deposition oxygen partial pressure. The film deposited at PO_*2*_ = 3 × 10^– 2^ mbar and 200 °C shows the highest subgap absorption, suggesting a relatively high density of localized states, likely associated with oxygen deficiency and structural disorder. However, further increasing the oxygen pressure to 6 × 10^–2^ and 10 × 10^–2^ mbar during ambient temperature deposition appears to reduce this effect, as evidenced by the superposition of the absorption spectra. This saturation behavior implies that beyond a certain oxygen threshold, additional oxygen incorporation may have limited influence on subgap state formation under low-temperature conditions. Differently, when the deposition temperature is at 200 °C, the saturation of the subgap absorption is less evident, with the sample with intermediate pressure presenting the lowest value of absorption. These trends highlight a complex relationship between the deposition temperature and oxygen availability in defect formation in MoO_*x*_ films.

#### 3.3.1 Optical Gap and Urbach Energy

The films were studied by using absorption spectra obtained through the PDS setup. From these spectra, the optical energy gap, *E*_opt_, can be obtained from the Tauc’s relation:^42^

1 $${\left(\alpha h\nu \right)}^{n}=B\left(h\nu -E_{\mathrm{opt}}\right)$$

where α is the absorption coefficient, *h*ν is the photon energy, *n* is a coefficient dependent on the material (1/2 for materials with an indirect bandgap, such as MoO_3_ and WO_3_),^43,44^ and *B* is a constant. The Tauc plots of the tungsten oxide samples deposited at ambient temperature and at 200 °C with PO_2_ = 10 × 10^–2^ mbar are presented in Figure S7, and the Tauc plots of the molybdenum oxide samples deposited at ambient temperature and at 200 °C with PO_2_ = 10 × 10^–2^ mbar are presented in Figure S8). In both figures, the red arrow represents graphically the linear regression with the intercept on the *x*-axis highlighting the estimated gap value.

Another parameter used to evaluate the quality of the films is the Urbach energy, *E*_u_, which can also be derived from the absorption coefficient:^42^

2 $$\alpha =\alpha_{0}\exp\left(\frac{h\nu }{E_{u}}\right)$$

where α_0_ is a constant.

The resulting values of the optical gaps and Urbach energies are presented in Table 2.

**Table 2:** Optical Gap and Urbach Energy of the WO_*x*_ and MoO_*x*_ Samples Deposited under Different Conditions

		WO_*X*_		MoO_*X*_	
PO_2_ (mbar)	*T*_dep_(°C)	*E*_opt_ (eV)	*E*_u_ (meV)	*E*_opt_ (eV)	*E*_u_ (meV)
3 × 10^–2^	*T*_amb_	3.20	158	3.05	187
6 × 10^–2^	*T*_amb_	3.21	112	3.16	159
10 × 10^–2^	*T*_amb_	3.29	137	3.15	142
3 × 10^–2^	200	3.16	143	2.75	423
6 × 10^–2^	200	3.20	175	3.00	220
10 × 10^–2^	200	3.12	139	2.92	235

The tungsten oxide samples exhibit an optical bandgap higher than 3.12 eV with the samples deposited at ambient temperature presenting a value above 3.2 eV. The samples deposited at 200 °C present a slightly lower optical band gap, which highlights a higher defectivity in the samples deposited at higher temperature. This trend is in accordance with the results of the PDS spectroscopy and the values of Urbach energy, which are generally higher, for the tungsten oxide samples deposited at 200 °C.

For the molybdenum oxide samples, the film deposited at PO_*2*_ = 3 × 10^– 2^ mbar and 200 °C shows the lowest optical bandgap and the highest Urbach energy, highlighting a relatively high density of localized states, likely associated with oxygen deficiency and structural disorder. The sample that exhibits the higher optical gap is the one deposited at PO_*2*_ = 6 × 10^– 2^ mbar at ambient temperature, and this sample presents also a relatively low value of Urbach energy when compared with the other MoO_*x*_ samples. These trends confirm the suggestion given by the PDS analysis in defect formation in MoO_*x*_ films due to temperature and oxygen pressure conditions.

### 3.4 Defect Density Evaluation

The absorption coefficient as measured by PDS is modeled using the one-electron approximation as expressed in eq 3, which assumes that each absorbed photon promotes an electron from an occupied state to an unoccupied one.^8^ To account for the observed subgap absorption peak in the near-infrared, an additional term representing small polaron absorption is included, which arises from localized electron–lattice.^8,9^ Polarons are quasi-particles used to model the interaction of trapped electrons with the surrounding atoms^45^ and are the cause of the peak in the near-infrared region of the absorption spectrum.^40,46,47^ Moreover, small polarons have previously been assessed in TMOs.^46,48,49^ Their contribution to absorption is modeled with an additive term in eq 3, expressing a weakly asymmetric Gaussian peak^46,47^ with a pre-exponential factor *A*_p_, polaron binding energy *E*_p_, and longitudinal-optical phonon energy *E*_op_:

3 $$\alpha \left(h\nu \right)=\frac{C}{h\nu }\int N_{i}\left(E\right)F\left(E\right)N_{f}\left(E+h\nu \right)\left[1-F\left(E+h\nu \right)\right]dE+\frac{A_{p}}{h\nu }e^{\left(-\frac{{\left(h\nu -2E_{p}\right)}^{2}}{8E_{p}E_{op}}\right)}$$

The photon energy is represented by *h*ν, while *C* is a parameter influenced by the refractive index and momentum matrix elements, assumed constant for all optical transitions;^50^ *N*_i_(*E*) represents the density of initial occupied states, *N*_f_(*E*) represents the density of final empty states, and *F*(*E*) is the Fermi–Dirac function. The constant *C* was determined for each spectrum using eq 3 at 4 eV, with the values provided in Tables 3 and 4 for the various WO_*x*_ and MoO_*x*_ samples. At this energy level, the optical transition from the valence band to the conduction band predominates; therefore, only this transition is considered for the calculation of *C*.

**Table 3:** C Constant, DOS, and Small Polaron Coefficients for the WO_*x*_ Samples Deposited under Different Conditions of Temperature and Pressure

PO_2_ (mbar)	*T*_dep_ (°C)	*C* (cm^5^·eV^2^)	*E*_0V_ (meV)	*E*_0C_ (meV)	*A*_D_ (eV^–1^ cm^–3^)	*E*_D_ (eV)	*W*_D_ (eV)	*A*_P_ (eV^–1^ cm^–3^)	*E*_P_ (eV)	*E*_op_ (meV)
3 × 10^–2^	*T*_amb_	1.61 × 10^–30^	134	62	1.96 × 10^13^	0.96	0.02	2.79 × 10^2^	0.35	0.29
6 × 10^–2^	*T*_amb_	1.69 × 10^–30^	101	81	1.48 × 10^13^	0.88	0.02	6.38 × 10^2^	0.38	0.27
10 × 10^–2^	*T*_amb_	4.59 × 10^–31^	92	56	3.25 × 10^12^	0.89	0.03	3.99 × 10^2^	0.44	0.23
3 × 10^–2^	200	1.46 × 10^–30^	126	82	1.94 × 10^13^	0.94	0.02	3.72 × 10^2^	0.37	0.27
6 × 10^–2^	200	1.70 × 10^–30^	110	84	8.65 × 10^13^	0.83	0.02	3.69 × 10^3^	0.35	0.29
10 × 10^–2^	200	1.33 × 10^–30^	127	45	1.24 × 10^14^	0.91	0.02	3.93 × 10^3^	0.35	0.29

**Table 4:** C Constant, DOS, and Small Polaron Coefficients for the MoO_*x*_ Samples Deposited under Different Conditions of Temperature and Pressure

PO_2_ (mbar)	*T*_dep_ (°C)	*C* (cm^5^ eV^2^)	*E*_0V_ (meV)	*E*_0C_ (meV)	*A*_D_ (eV^–1^ cm^–3^)	*E*_D_ (eV)	*W*_D_ (eV)	*A*_P_ (eV^–1^ cm^–3^)	*E*_P_ (eV)	*E*_op_ (meV)
3 × 10^–2^	*T*_amb_	7.19 × 10^–32^	137	88	1.97 × 10^15^	1.20	0.05	7.09 × 10^4^	0.41	1.54
6 × 10^–2^	*T*_amb_	8.41 × 10^–32^	550	51	1.93 × 10^15^	1.25	0.06	4.59 × 10^3^	0.48	1.89
10 × 10^–2^	*T*_amb_	1.25 × 10^–31^	107	54	1.53 × 10^15^	1.27	0.08	6.46 × 10^3^	0.47	1.91
3 × 10^–2^	200	6.23 × 10^–32^	200	94	2.89 × 10^16^	1.20	0.04	2.82 × 10^5^	0.38	1.47
6 × 10^–2^	200	1.28 × 10^–31^	119	59	3.34 × 10^13^	1.10	0.06	3.59 × 10^2^	0.40	1.56
10 × 10^–2^	200	1.11 × 10^–31^	137	46	4.34 × 10^14^	1.01	0.08	2.67 × 10^3^	0.43	1.67

The density of states in eq 3 can be modeled through the sum of several distributions as in eq 4 with a parabolic trend for valence and conduction bands (*N*_VB_, *N*_CB_), an exponential trend for the band tails (*N*_VBT_, *N*_CBT_), and a Gaussian distribution for localized midgap defect states (*N*_D_).^8,9,12,50^

4 $$DOS(E)\to \{\begin{array}{ll} N_{VB}(E)=N_{V}\sqrt{‐E+E_{G}+E_{0V}} & E<0\\ N_{CB}(E)=N_{C}\sqrt{E‐E_{0C}} & E\geq 0\\ N_{VBT}(E)=N_{V}\sqrt{\frac{E_{0V}}{2}}\exp(‐\frac{E+E_{G}}{E_{0V}}) & E>E_{G}\\ N_{CBT}(E)=N_{C}\sqrt{\frac{E_{0C}}{2}}\exp(\frac{E}{E_{0C}}) & E<0\\ N_{D}(E)=\frac{A_{D}}{\sqrt{2\pi W_{D}^{2}}}\exp\left[‐\frac{1}{2}{\left(\frac{E+E_{D}}{W_{D}}\right)}^{2}\right] & E_{G}<E<0 \end{array}$$

Here, the energy reference level was considered at the conduction band limit; the density of states of the valence and conduction bands are *N*_V_, and *N*_C_, respectively. The slopes of the valence and conduction band tails are *E*_0V_ and *E*_0C_, and the bandgap is *E*_G_. The defects are modeled by the area of the Gaussian distribution *A*_D_, the mean *E*_D,_ and the fwhm *W*_D_.

For the tungsten oxide samples, the results are given in Table 3. The valence band slope *E*_0V_ shows a mean value of 117 meV with a peak of 134 meV for the sample deposited at room temperature at PO_2_ = 3 × 10^–2^ mbar. Similarly, *E*_0C_ shows a mean value of 68 meV with a peak of 84 meV for the sample deposited at 200 °C and 6 × 10^–2^ mbar. It should be noted that the samples deposited at room temperature with PO_2_ = 3 × 10^–2^ and 6 × 10^–2^ mbar show a slightly lower value than the ones deposited at 200 °C with the same pressure. Regarding the parameters of the defect distribution, the *E*_D_ is on average 0.9 eV below the conduction band edge, with standard deviations within 20 meV. This energy position agrees with other studies, which connected this defect to oxygen vacancies.^51^ The defects area, *A*D, is also reported in Figure 6a where it can be seen that, for each deposition pressure, the sample grown at a higher temperature always shows a higher value; moreover, there is an ascending trend as the pressure rises. Figure 6b shows the data extrapolated from the fitting relative to the polaron factor *A*_p_. A similar trend to the area of the defects is observed by increasing oxygen pressure at 200 °C, whereas at room temperature, *A*_p_ decreases with increasing oxygen pressure. Finally, *E*_P_ and *E*_op_ show low variation for different conditions with mean values of 0.37 eV and 0.27 meV.

**Figure 6:**
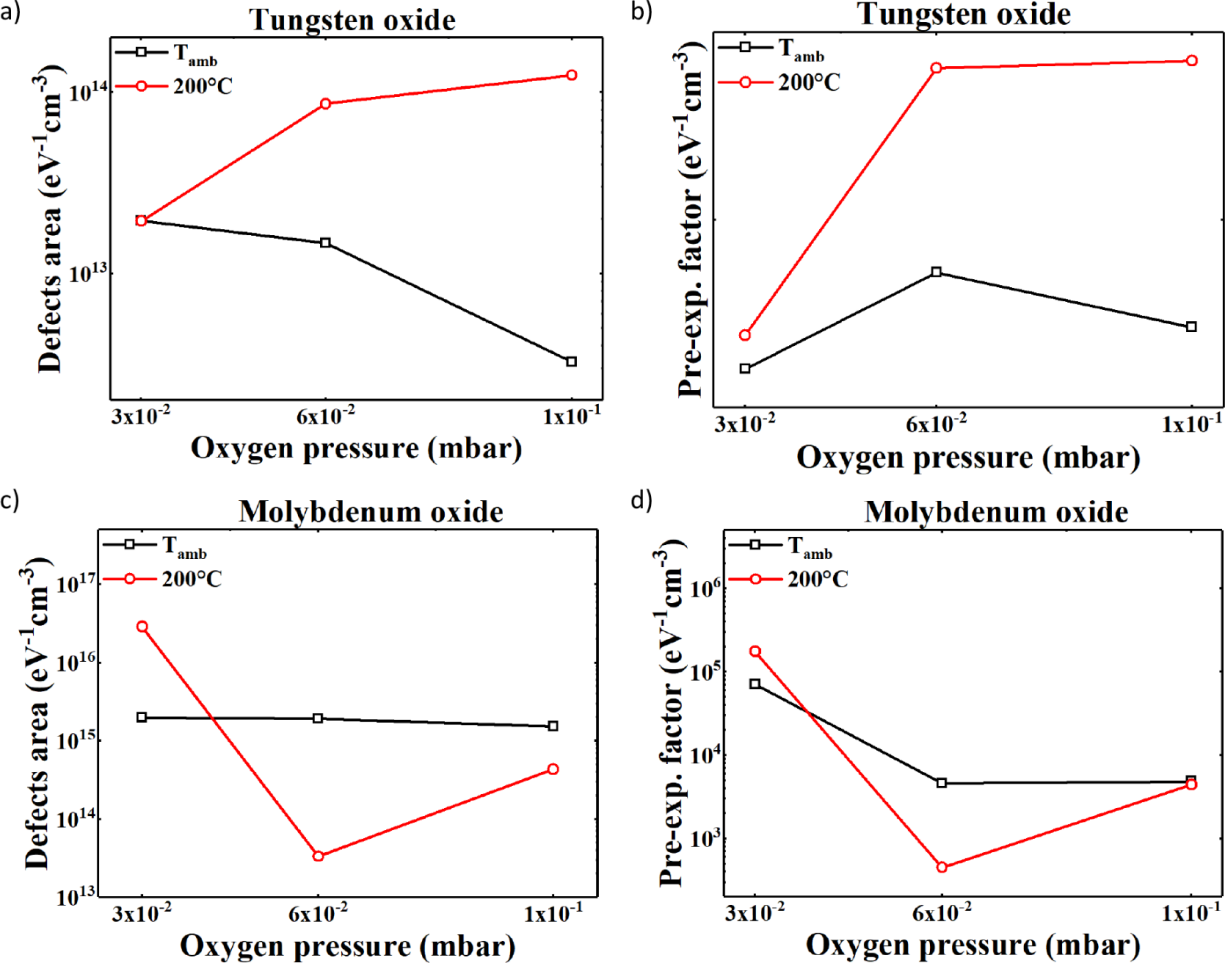
Resulting parameters for the samples under different deposition conditions: WO_*x*_ (a) *A*_D_ and (b) *A*_p_; MoO_*x*_ (c) *A*_D_ and (d) *A*_p_.

For the molybdenum oxide samples, the results are listed in Table 4. The valence band slope *E*_0V_ shows a mean value of 208 meV with a peak of 550 meV for the sample deposited at ambient temperature and 6 × 10^–2^ mbar. Except for the sample deposited with 3 × 10^–2^ mbar, the sample at room temperature shows a value that is slightly lower than the samples deposited at 200 °C. Similarly, *E*_0C_ shows a mean value of 64 meV with a peak of 94 meV for the sample deposited at 200 °C and 3 × 10^–2^ mbar. It should be noted that all of the samples deposited at room temperature show a value that is slightly lower than the samples deposited at 200 °C except for the sample deposited with 10 × 10^–2^ mbar. Regarding the parameters of the defect distribution, the *E*_D_ is on average 1.2 eV below the conduction band edge, with standard deviations within 60 meV. This energy position agrees with other studies that linked these defects to the oxygen vacancies.^52^ The defect area is also reported in Figure 6c where a small variation is shown for the samples deposited at room temperature, while at higher temperatures, there is a descending trend as the oxygen pressure rises. Figure 6d presents the data obtained from fitting the *A*_p_ factor. This parameter shows a trend similar to that of one of the defect areas for rising PO_2_ and temperature. Lastly, *E*_P_ and *E*_op_ show low variation for different conditions with mean values of 0.43 eV and 1.68 meV.

Although direct electrical characterization was not provided, the defect density evaluation obtained through PDS provides indirect insights into the charge transport limitations. Higher defect densities correlate with increased carrier trapping, which likely contributes to the observed transport behavior of these films. This highlights the importance of optimizing the stoichiometry and deposition conditions to minimize defect-related recombination losses.

To further illustrate the distribution of defect states in MoO_*x*_ and WO_*x*_, Figure 7 presents the density of states of the samples deposited at room temperature with 6 × 10^–2^ mbar. Figure 7 also shows the single contribution for each distribution: the valence band and band-tail, the Gaussian defect states within the bandgap, and the conduction band and band-tail. This diagram provides a visual interpretation of the absorption-derived density of states, highlighting the role of oxygen vacancies in subgap absorption and charge trapping. The density of states is positioned relative to the conduction band limit.

**Figure 7:**
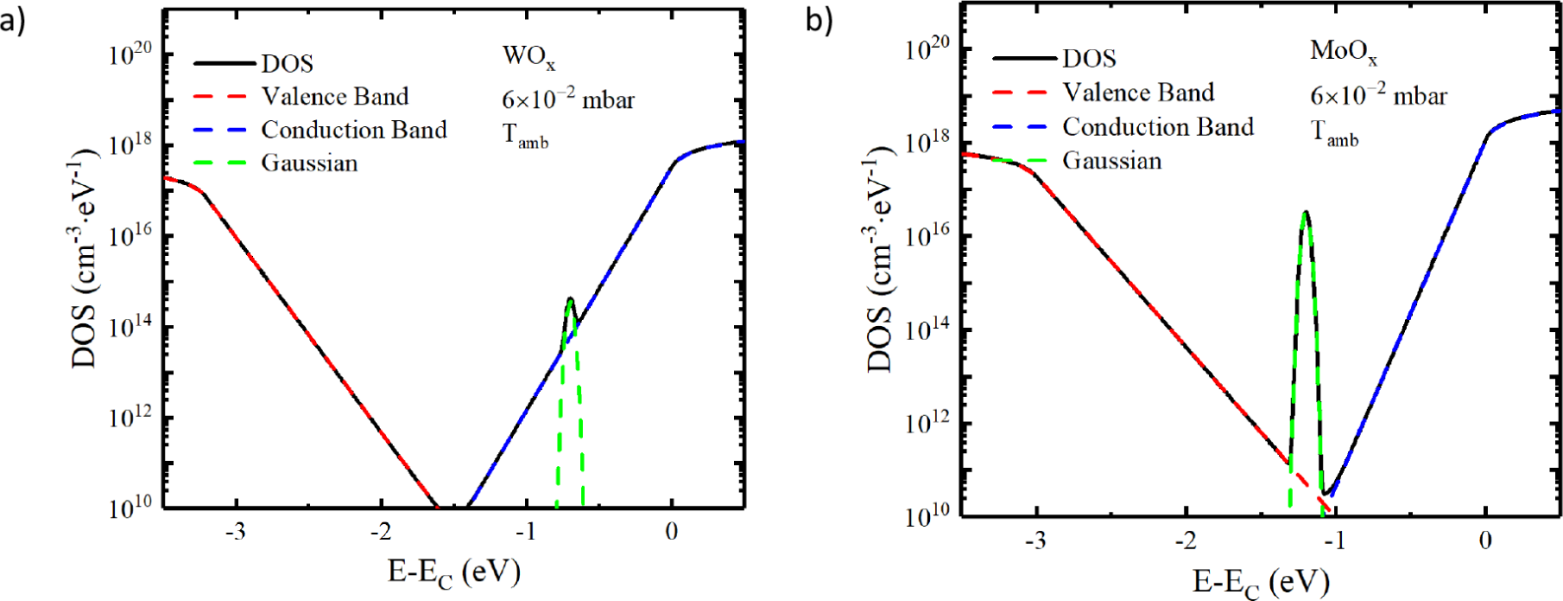
Resulting DOS (black line) with Gaussian defect distributions, the valence, conduction bands, and tails for the samples deposited at ambient temperature and 6 × 10^–2^ mbar: (a) WO_*x*_ and (b) MoO_*x*_.

The defectivity of the samples is quantitatively represented by the parameter *A*_D_, which corresponds to the area under the Gaussian defect distribution. This area is directly related to the overall defect density, while the other parameters *E*_D_ and *W*_D_ describe the energy location and spread of the defect states within the bandgap. A comparative analysis of the fitted *A*_D_ values is shown in Figure 6a for the tungsten oxide samples and in Figure 6b for the molybdenum oxide samples.

For tungsten oxide, the samples deposited at 200 °C consistently exhibit a higher defect density than their room-temperature counterparts. A clear trend of increasing *A*_D_ with increasing oxygen pressure is observed at 200 °C, whereas the room-temperature samples show the opposite behavior. The sample deposited at 200 °C and PO_2_ = 10 × 10^– 2^ mbar shows the highest defectivity, with an *A*_D_ of 1.24 × 10^14^ eV^–1^ cm^–3^, while the corresponding room-temperature sample has the lowest value, an *A*_D_ of 3.25 × 10^12^ eV^–1^ cm^–3^. These trends are consistent with the qualitative differences observed in the PDS-measured absorption spectra.

In the case of molybdenum oxide, the defect density of the ambient-temperature samples is relatively insensitive to increasing PO_2_, while for the 200 °C samples, *A*_D_ decreases significantly with a higher oxygen pressure. The most defective sample was deposited at 200 °C with PO_*2*_ = 3 × 10^–2^ mbar, exhibiting *A*_D_ = 2.89 × 10^16^ eV^–1^ cm^–3^. The lowest value among the MoO_*x*_ samples is found in the 200 °C sample deposited at PO_2_ = 6 × 10^–2^ mbar, with *A*_D_ = 3.34 × 10^13^ eV^–1^ cm^–3^. These findings are again in agreement with the qualitative PDS trends, confirming that the model accurately captures the impact of processing conditions on subgap defect formation.

### 3.5 Solar Cell Prototypes Study

The *J*–*V* curve characteristics experimentally evaluated under AM 1.5 illumination for the heterojunction solar cell prototypes with transition-metal oxides as selective carrier contacts present an S-shaped *J*–*V* characteristic. It should be noted that the oxide layers used in the solar cell prototypes were deposited at ambient temperature. This limitation is due to the use of hydrogenated amorphous silicon (a-Si:H) in the device architecture, which has a low thermal budget of approximately 130 °C. Higher deposition temperatures, such as 400 °C, would lead to hydrogen effusion and partial crystallization of the a-Si:H layer, compromising its passivation quality and the overall performance of the heterojunction solar cell.

The experimental *J–V* characteristics of the cells with tungsten and molybdenum oxide hole-selective contact are presented in Figure 8 to show the S-shape behavior for the cell implementing the TMO hole-selective contact. The observed S-shaped *J*–*V* curves suggest a barrier to charge extraction, likely resulting from misalignment between the band structures of TMOs and hydrogenated amorphous silicon, as discussed in previous studies.^23,53^

**Figure 8:**
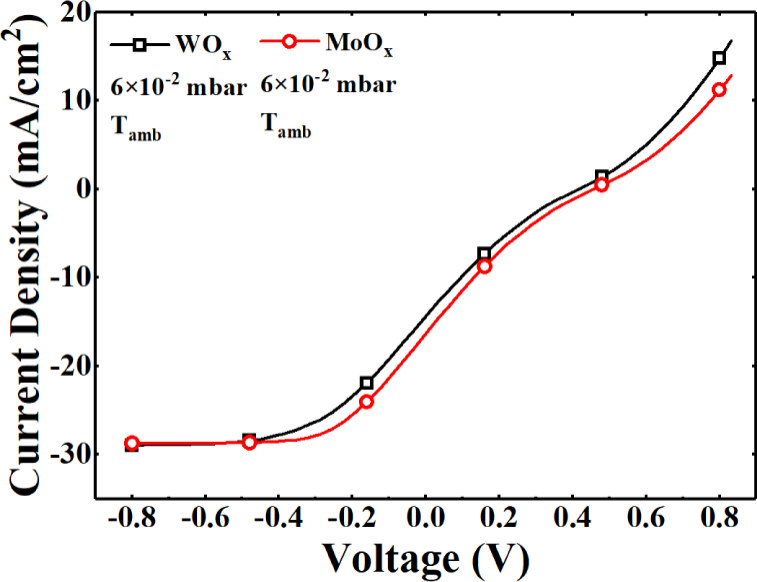
Experimental *J*–*V* curves of a TMO-based HJT solar cell showing an S-shape curve under AM 1.5. The black line with squares is referred to WO_*X*_, while the red line with circles is referred to MoO_*x*_.

The charge extraction barrier indicated by S-shaped *J*–*V* curves can be attributed to band misalignment and defect-assisted recombination. In particular, subgap absorption from oxygen vacancies introduces additional carrier traps, which contribute to recombination losses. Addressing these limitations may require interface engineering strategies, such as optimizing the TMO stoichiometry or introducing buffer layers to facilitate smoother band transitions.

As observed in the defect density analysis, substoichiometric films exhibit increased Urbach energy, which correlates with higher defect densities. These findings suggest that further optimization of the stoichiometry and interface engineering is necessary to mitigate these losses.

This phenomenon brings limitations in the optimal design of transition-metal-oxide-based heterojunction solar cells. Parameters like the optical band gap, work function, crystallinity, and stoichiometry depend on the deposition technique, substrate temperature, and oxygen pressure, potentially leading to nonoptimal electrical characteristics.

The equivalent electrical model of a heterojunction solar cell is typically based on p*–*n junctions. However, the presence of an S-shaped *J*–*V* characteristic cannot be accurately modeled using this approach. For including these anomalous S-shape DC electrical characteristics, different models exist in the literature.^12,54,55^ For this study, we adopted the equivalent electrical model as depicted in Figure 9 and developed in the literature.^56,57^ In this model, a rectifying Schottky junction (SJ) is connected in series with the heterojunction to introduce a Schottky barrier.

**Figure 9:**
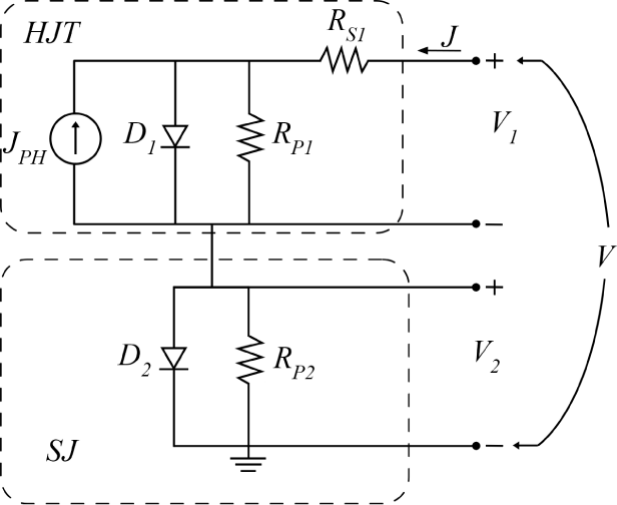
Equivalent electrical model of an HJT solar cell including a SJ.

The S-shaped observation in the *J*–*V* characteristics indicates the presence of nonideal charge extraction mechanisms. This behavior can be attributed to a combination of interfacial barriers and recombination processes. The factors that could lead to such a behavior are (i) incomplete or unfavorable energy level alignment between the TMO and the adjacent layers, as it can create a transport barrier for holes, especially if the TMO work function is too low or not matched to the valence band of a-Si:H­(i); (ii) the presence of defect states at the TMO/a-Si:H­(i) interface, as it can facilitate nonradiative recombination; and (iii) the band bending at the TMO/a-Si:H­(i) interface, as it can result in band misalignment that opposes carrier extraction.

This is effectively modeled in the presented equivalent circuit via a barrier element in series with the main junction, which allows reproducing the observed S-shaped curve. These effects may act simultaneously with varying dominance depending on the deposition conditions.

The equivalent model of the solar cell is described by the following equations:

5 $$J=J_{01}\left(\exp\left(\frac{V_{1}-JR_{S1}}{n_{1}V_{T}}\right)-1\right)+\frac{V_{1}-JR_{S1}}{R_{P1}}-J_{PH}$$

6 $$J=J_{02}\left(\exp\left(\frac{V_{2}}{n_{2}V_{T}}\right)-1\right)+\frac{V_{2}}{R_{P2}}$$

7 $$V=V_{1}+V_{2}$$

where the current density through the solar cell is denoted as *J* and the bias voltage of the cell is *V*. For the HJT, the bias voltage is *V*_1_, the dark saturation current density is *J*_01_, the ideal factor is *n*_1_, the thermal voltage is *V*_T_, the *J*_PH_ is the photogenerated current density, and *R*_*S*1_ and *R*_*P*1_ are the series resistance and shunt resistance, respectively. Regarding the SJ, the leakage current density is *J*_02_, the bias voltage is *V*_2_, the ideal factor is *n*_2_, and the shunt resistance is *R*_*P*2_. The derivation of *V*_2_ as a function of *J* is obtained through the Lambert *W* function as expressed in eq 8.

8 $$V_{2}=R_{P2}\left(J_{02}+J\right)+n_{2}V_{T}W\left(\frac{J_{02}R_{P2}}{n_{2}V_{T}}\exp\left(\frac{R_{P2}\left(J_{02}+J\right)}{n_{2}V_{T}}\right)\right)$$

The model presented in Figure 9 and in eqs 5–8 allows for an interpretation of the *J*–*V* characteristics observed in the devices affected by the S-shape. The diode *D*_1_ is for describing the ideal behavior of the junction, while the resistor *R*_S1_ reflects the resistive losses due to carrier transport in the solar cell stack and contacts. The shunt resistors, which are *R*_P1_ and *R*_P2_, account for leakage pathways and interface recombination; both of these leakages can be of particular significance in devices with a high defect density. The second diode *D*_2_ is used to address the bias-voltage-dependent carrier recombination by modeling the rectifying properties induced by the Schottky barrier, interfacial dipole, and unbalanced charge transport.^56^ The ideality factor *n*_2_ of the Schottky diode is usually large and it means that the effective recombination lifetime in the depletion layer increases with an increasing recombination rate due to trap-assisted tunneling or field-enhanced recombination via single levels or even flowing at the edge of the diode and in certain shunt positions.^12,58^

The previous parameters were obtained by fitting the *JV* curves of the prototypes to the model. The resulting parameters allow for an in-depth analysis and comprehension of the device and its underlying mechanisms. Figure 10 shows a comparison of the dark saturation current *J*_0_ for solar cells based on the two different TMOs investigated above. It can be noted that the highest value of *J*_0_ is obtained for both WO_*x*_ and MoO_*x*_ hole contacts deposited with the lowest oxygen pressure. As PO_2_ increases, *J*_0_, for both TMOs, is reduced by an order of magnitude.

**Figure 10:**
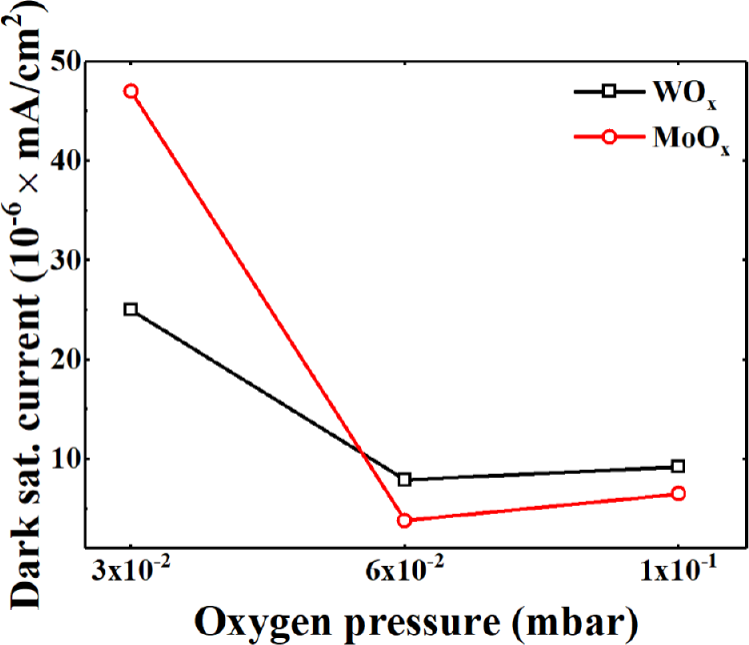
Dark saturation current vs oxygen pressure of the prototypes of solar cells.

Additionally, a clear correlation is observed between the subgap defect density, quantified by both the defect area and the Urbach energy, and the electrical performance of the devices.

For both TMOs, defectivity and Urbach energy decrease with increasing oxygen pressure, which corresponds to improved electrical parameters (higher short-circuit current density *J*_SC_ and open-circuit voltage *V*_OC_). In contrast, MoO_*x*_ samples show a more complex behavior, leading to less predictable trends. Nonetheless, the highest open-circuit voltage is obtained with the lowest oxygen deposition pressure. Interestingly, in both TMO-based solar cells, an increase in localized defect states appears to coincide with improved electrical characteristics, suggesting the possible involvement of trap-assisted tunneling mechanisms that enhance hole extraction. However, excess defect density can also increase recombination losses, highlighting the need for a careful balance between improving transport and minimizing recombination. These results emphasize that optimizing the defect within TMOs is crucial for achieving optimal energy level alignment and efficient carrier extraction in heterojunction solar cells.

Finally, a simulation of the electrical characteristics, including only the HJT without the SJ, is presented in Figure 11a,b for WO_*x*_ and MoO_*x*_ hole contacts, respectively, deposited under the oxygen pressure that corresponds to the lower value of *J*_0_. The *J–V* curves of Figure 11 share the same *V*_OC_ of experimental *J–V*. When the current is zero, the total voltage drop across the cell is given by the section of the equivalent circuit model referred to as the heterojunction cell. For the negative bias voltage, the current density is the photogenerated current density. For positive bias voltage, the slope of the *J*–*V* curve is slightly higher for the sample with the hole-selective contact made of molybdenum oxide. The characteristic presented in Figure 11 forecasts the behavior of the solar cells implementing the doping-free hole-selective contact without the presence of the barrier introduced by a mismatch in the band structure of the device between the layers of TMO and the a-Si:H­(i). Such a simulation is retrieved considering only the section of the model with the heterojunction parameters and zeroing in on the Schottky region voltage drop.

**Figure 11:**
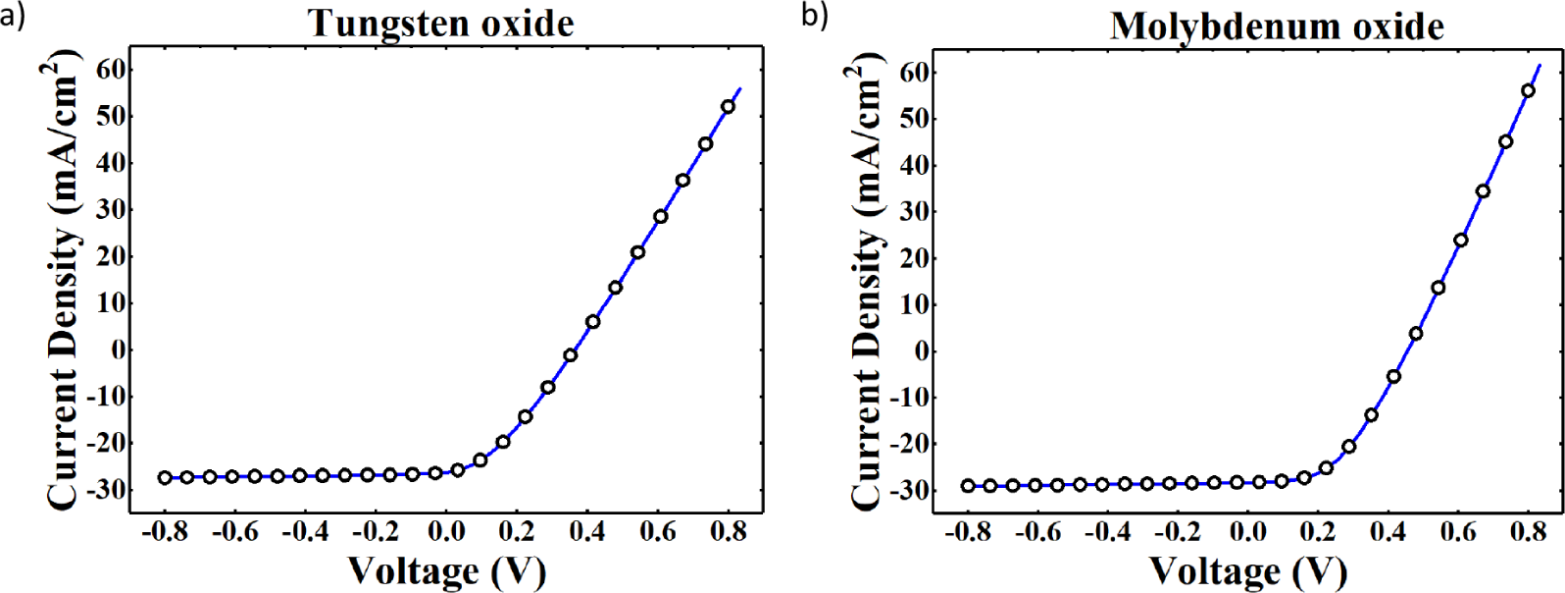
Simulation of the electrical characteristic of the solar cells prototype with hole contacts based on (a) WO_*x*_ deposited at an oxygen pressure of 6 × 10^–2^ mbar and (b) MoO_*x*_ deposited at an oxygen pressure of 10 × 10^–2^ mbar.

## 4 Conclusions

This study presented a comprehensive analysis of substoichiometric MoO_*x*_ and WO_*x*_ thin films deposited by pulsed laser deposition as hole-selective contacts for silicon heterojunction solar cells. While these materials offer potential for doping-free carrier-selective contacts, their performance is influenced by defect states that contribute to charge extraction barriers and the emergence of S-shaped *J*–*V* characteristics.

To investigate these limitations, we performed combined structural, optical, and electrical analyses using X-ray photoelectron spectroscopy, Raman spectroscopy, photothermal deflection spectroscopy, and *J*–*V* characterization of solar cell prototypes. PDS revealed that defect-related subgap absorption is closely tied to deposition temperature and oxygen partial pressure, with higher oxygen content generally reducing the defect density. The Gaussian defect area and Urbach energy extracted from the density-of-states modeling provided a semiquantitative link between material properties and device metrics. Devices with higher defectivity exhibited an increased saturation current density and open-circuit voltage, consistent with enhanced trap-assisted tunneling at the TMO/a-Si:H­(i) interface.

The observed S-shaped *J*–*V* characteristics were further analyzed using an equivalent circuit model, which revealed that a combination of interface recombination, back-barrier formation, and defect-induced transport limitations contribute to the nonideal behavior. These findings highlight that deposition conditions, particularly oxygen pressure and substrate temperature, play a critical role in determining the defect density, energy level alignment, and charge transport behavior of these transition metal oxides.

Future work should focus on direct experimental measurements of key electronic properties, such as work function and carrier transport, using techniques such as Kelvin probe and transfer length method measurements. Besides, alternative deposition techniques could be evaluated for scalability and industrial feasibility. Finally, interface engineering strategies, including passivation layers or doping schemes, should be investigated to tune the work function of the TMOs, optimizing the band alignment and reducing the recombination losses.

Although this study focuses on hole-selective contacts for silicon heterojunction solar cells, transition metal oxides play an active role in other functional energy applications, such as photocatalysis and electrocatalysis, where these results may also have relevance.

## Supplementary Material



## Notes

Additional details regarding the PLD parameters and deposition conditions and additional experimental results such as XPS and Tauc plot (PDF)

## References

[1] Simon Philipps; Werner Warmuth. Photovoltaics Report; Freiburg, 2024 https://www.ise.fraunhofer.de/content/dam/ise/de/documents/publications/studies/Photovoltaics-Report.pdf. accessed 18 April 2025.

[2] International Technology for Photovoltaics (ITRPV), Sixteenth Edition; 2025 https://www.vdma.org/international-technology-roadmap-photovoltaic. accessed 18 April 2025.

[3] LONGi. LONGi Sets New World-Record for Silicon Solar Cell Efficiency, Launching 2nd Generation Ultra-Efficient BC-Based Module https://www.longi.com/en/news/longi-hi-mo9-bc-world-record/. accessed 19 April 2025.

[4] Green M. A., Dunlop E. D., Yoshita M., Kopidakis N., Bothe K., Siefer G., Hao X. (2024). Solar Cell
Efficiency
Tables (Version 63). Prog. Photovolt: Res. Appl..

[5] Liu W., Meng F., Zhang X., Liu Z. (2015). Evolution of a Native
Oxide Layer at the A-Si: H/c-Si Interface and Its Influence on a Silicon
Heterojunction Solar Cell. ACS Appl. Mater.
Interfaces.

[6] Li J., Pan T., Wang J., Cao S., Lin Y., Hoex B., Ma Z., Lu L., Yang L., Sun B., Li D. (2020). Bilayer MoOX/CrOX
Passivating Contact Targeting Highly Stable Silicon Heterojunction
Solar Cells. ACS Appl. Mater. Interfaces.

[7] Mews M., Lemaire A., Korte L. (2017). Sputtered Tungsten Oxide as Hole
Contact for Silicon Heterojunction Solar Cells. IEEE J. Photovoltaics.

[8] Scirè D., Procel P., Gulino A., Isabella O., Zeman M., Crupi I. (2020). Sub-Gap Defect Density
Characterization of Molybdenum Oxide: An Annealing
Study for Solar Cell Applications. Nano Res..

[9] Scirè D., Macaluso R., Mosca M., Mirabella S., Gulino A., Isabella O., Zeman M., Crupi I. (2021). Characterization
of the Defect Density States in MoOx for C-Si Solar Cell Applications. Solid-State Electron..

[10] Ahmadpour M., Fernandes Cauduro A. L., Méthivier C., Kunert B., Labanti C., Resel R., Turkovic V., Rubahn H. G., Witkowski N., Schmid A. K., Madsen M. (2019). Crystalline Molybdenum Oxide Layers
as Efficient and Stable Hole Contacts in Organic Photovoltaic Devices. ACS Appl. Energy Mater..

[11] Yin X., Battaglia C., Lin Y., Chen K., Hettick M., Zheng M., Chen C.-Y., Kiriya D., Javey A. (2014). 19 2. Efficient
InP Heterojunction Solar Cell with Electron-Selective TiO2 Contact. ACS Photonics.

[12] Scirè D., Macaluso R., Mosca M., Casaletto M. P., Isabella O., Zeman M., Crupi I. (2022). Density of
States Characterization
of TiO2 Films Deposited by Pulsed Laser Deposition for Heterojunction
Solar Cells. Nano Res..

[13] Koshi N. A., Murthy D. H. K., Chakraborty S., Lee S. C., Bhattacharjee S. (2022). Probing Photoexcited
Charge Carrier Trapping and Defect Formation in Synergistic Doping
of SrTiO3. ACS Appl. Energy Mater..

[14] Yamada I., Takamatsu A., Asai K., Ohzuku H., Shirakawa T., Uchimura T., Kawaguchi S., Tsukasaki H., Mori S., Wada K., Ikeno H., Yagi S. (2018). Synergistically
Enhanced Oxygen Evolution Reaction Catalysis for Multielement Transition-Metal
Oxides. ACS Appl. Energy Mater..

[15] Horynová E., Romanyuk O., Horák L., Remeš Z., Conrad B., Peter Amalathas A., Landová L., Houdková J., Jiříček P., Finsterle T. (2020). Optical Characterization of Low Temperature Amorphous
MoOx, WOX, and VOx Prepared by Pulsed Laser Deposition. Thin Solid Films.

[16] Hao L. C., Zhang M., Ni M., Liu J. M., Feng X. D. (2018). Simulation
of High Efficiency Silicon Heterojunction Solar Cells with Molybdenum
Oxide Carrier Selective Layer. Mater. Res. Express.

[17] Zhao Y., Mazzarella L., Procel P., Han C., Yang G., Weeber A., Zeman M., Isabella O. (2020). Doped Hydrogenated
Nanocrystalline Silicon Oxide Layers for High-Efficiency c-Si Heterojunction
Solar Cells. Prog. Photovolt: Res. Appl..

[18] Spinelli P., Sen M. A., Hoek E. G., Kikkert B. W. J., Yang G., Isabella O., Weeber A. W., Bronsveld P. C. P. (2018). Moly-Poly
Solar Cell: Industrial Application of Metal-Oxide Passivating Contacts
with a Starting Efficiency of 18.1%. AIP Conf.
Proc..

[19] Cao L., Procel P., Alcañiz A., Yan J., Tichelaar F., Özkol E., Zhao Y., Han C., Yang G., Yao Z., Zeman M., Santbergen R., Mazzarella L., Isabella O. (2023). Achieving 23.83% Conversion Efficiency
in Silicon Heterojunction
Solar Cell with Ultra-Thin MoOx Hole Collector Layer via Tailoring
(i)­a-Si: H/MoOx Interface. Prog. Photovolt:
Res. Appl..

[20] Sun S., Xu M., Zhang Y., Liu R., Wang X., Zhang L., Fang Y., Wang P. (2023). Study of Molybdenum
Oxide Optimized
Hole Carrier Transport in Perovskite Solar Cells. Org. Electron..

[21] Messmer C., Bivour M., Schön J., Hermle M. (2018). Requirements for Efficient
Hole Extraction in Transition Metal Oxide-Based Silicon Heterojunction
Solar Cells. J. Appl. Phys..

[22] Messmer C., Bivour M., Schon J., Glunz S. W., Hermle M. (2018). Numerical
Simulation of Silicon Heterojunction Solar Cells Featuring Metal Oxides
as Carrier-Selective Contacts. IEEE J. Photovoltaics.

[23] Saive R. (2019). S-Shaped Current-Voltage
Characteristics in Solar Cells: A Review. IEEE
J. Photovoltaics.

[24] Zhong H., Zhou R., Wu X., Lin X., Wang Y., Li Q., Zhou H. (2021). Investigation of the
S-Shaped Current–Voltage
Curve in High Open-Circuit Voltage Ruddlesden–Popper Perovskite
Solar Cells. Front. Energy Res..

[25] Lupo F. V., Scirè D., Mosca M., Crupi I., Razzari L., Macaluso R. (2021). Custom Measurement
System for Memristor Characterisation. Solid-State
Electron..

[26] Larciprete M. C., Ceneda D., Scirè D., Mosca M., Adorno D. P., Dereshgi S. A., Macaluso R., Li Voti R., Sibilia C., Cesca T. (2023). Tunable
IR Perfect Absorbers Enabled by Tungsten Doped
VO2 Thin Films. APL Mater..

[27] Bile A., Ceneda D., Maryam V. E. S., Scirè D., Buscarino G., Mosca M., Adorno D. P., Macaluso R., Voti R. L., Sibilia C., Folland T. G., Aydin K., Centini M., Larciprete M. C. (2024). Room-Temperature Tuning of Mid-Infrared
Optical Phonons and Plasmons in W-Doped VO2 Thin Films. Opt. Mater..

[28] Naumkin, A. V. ; Kraust-Vass, A. ; Gaarenstroom, S. W. ; Powell, C. J. NIST X-ray Photoelectron Spectroscopy Database, National Institute of Standards and Technology; Measurement Services Division of the National Institute of Standards, NIST Standard Reference Database Number 20.

[29] Singh M., Santbergen R., Mazzarella L., Madrampazakis A., Yang G., Vismara R., Remes Z., Weeber A., Zeman M., Isabella O. (2020). Optical Characterization
of Poly-SiOx
and Poly-SiCx Carrier-Selective Passivating Contacts. Sol. Energy Mater. Sol. Cells.

[30] Bivour M., Temmler J., Steinkemper H., Hermle M. (2015). Molybdenum and Tungsten
Oxide: High Work Function Wide Band Gap Contact Materials for Hole
Selective Contacts of Silicon Solar Cells. Sol.
Energy Mater. Sol. Cells.

[31] Halek G., Baikie I. D., Teterycz H., Halek P., Suchorska-Woźniak P., Wiśniewski K. (2013). Work Function
Analysis of Gas Sensitive WO3 Layers
with Pt Doping. Sens. Actuators, B.

[32] Wang Z., Liu Z., Lin H., Ye F., Gao P. (2023). Hot Wire Oxidation–Sublimation
Derived Work Function Tunable WOx Thin Films for Building Hole-Selective
Contacts. Mater. Today Energy.

[33] Fang L., Baik S. J., Kim J. W., Kang S. J., Seo J. W., Jeon J. W., Kim Y. H., Lim K. S. (2011). Tunable Work Function
of a WOx Buffer Layer for Enhanced Photocarrier Collection of Pin-Type
Amorphous Silicon Solar Cells. J. Appl. Phys..

[34] Cauduro A. L. F., dos Reis R., Chen G., Schmid A. K., Rubahn H. G., Madsen M. (2017). Work Function Mapping
of MoOx Thin-Films for Application
in Electronic Devices. Ultramicroscopy.

[35] Schulz P., Tiepelt J. O., Christians J. A., Levine I., Edri E., Sanehira E. M., Hodes G., Cahen D., Kahn A. (2016). High-Work-Function
Molybdenum Oxide Hole Extraction Contacts in Hybrid Organic-Inorganic
Perovskite Solar Cells. ACS Appl. Mater. Interfaces.

[36] Mews M., Korte L., Rech B. (2016). Oxygen Vacancies
in Tungsten Oxide
and Their Influence on Tungsten Oxide/Silicon Heterojunction Solar
Cells. Sol. Energy Mater. Sol. Cells.

[37] Marot L., Fleury J., Haas D., Iyyakkunnel S., Sanchez F., Steiner R., Mathys D., Antunes R., Meyer E. (2022). Situ Work Function Measurements Of
W, WO3 Nanostructured Surfaces. Surf. Coat.
Technol..

[38] Zou Y. S., Zhang Y. C., Lou D., Wang H. P., Gu L., Dong Y. H., Dou K., Song X. F., Zeng H. B. (2014). Structural
and Optical Properties of WO3 Films Deposited by Pulsed Laser Deposition. J. Alloys Compd..

[39] De
Wijs G. A., De Groot R. A. (2001). Amorphous WO3: A First-Principles
Approach. Electrochim. Acta.

[40] Dieterle M., Weinberg G., Mestl G. (2002). Raman Spectroscopy
of Molybdenum
Oxides - Part I. Structural Characterization of Oxygen Defects in
MoO3-x by DR UV/VIS, Raman Spectroscopy and X-Ray Diffraction. Phys. Chem. Chem. Phys..

[41] Dieterle M., Mestl G. (2002). Raman Spectroscopy
of Molybdenum Oxides: Part II. Resonance Raman
Spectroscopic Characterization of the Molybdenum Oxides Mo4O11 and
MoO2. Phys. Chem. Chem. Phys..

[42] Klein J., Kampermann L., Mockenhaupt B., Behrens M., Strunk J., Bacher G. (2023). Limitations
of the Tauc Plot Method. Adv. Funct. Mater..

[43] Cárdenas R., Torres J., Alfonso J. E. (2005). Optical
Characterization of MoO3
Thin Films Produced by Continuous Wave CO2 Laser-Assisted Evaporation. Thin Solid Films.

[44] Subrahmanyam A., Karuppasamy A. (2007). Optical and
Electrochromic Properties of Oxygen Sputtered
Tungsten Oxide (WO3) Thin Films. Sol. Energy
Mater. Sol. Cells.

[45] Gulino A., Tabbì G. (2005). CdO Thin Films: A Study of Their
Electronic Structure
by Electron Spin Resonance Spectroscopy. Appl.
Surf. Sci..

[46] Ederth J., Hoel A., Niklasson G. A., Granqvist C. G. (2004). Small Polaron
Formation in Porous WO3-x Nanoparticle Films. J. Appl. Phys..

[47] Niklasson G. A., Klasson J., Olsson E. (2001). Polaron Absorption
in Tungsten Oxide
Nanoparticle Aggregates. Electrochim. Acta.

[48] Reticcioli, M. ; Diebold, U. ; Kresse, G. ; Franchini, C. Small Polarons in Transition Metal Oxides. In Handbook of Materials Modeling, Andreoni, W. ; Yip, S. , Eds.; Springer: Cham, 2019, pp. 1–39. DOI: 10.1007/978-3-319-50257-1_52-1.

[49] Nitharwal R. K., Sahoo A., Kumar V., Rao M. S. R., Dixit T., Krishnan S. (2025). Spectroscopic Visualization
of Polarons and Intervalence
Charge Transfer in MoO _3– *x*
_ Nanostructures Via Defect Engineering. ACS
Mater. Lett..

[50] Bouizem Y., Belfedal A., Sib J. D., Chahed L. (2003). Density of States in
Hydrogenated Amorphous Germanium Seen via Optical Absorption Spectra. Solid State Commun..

[51] Gerosa M., Bottani C. E., Caramella L., Onida G., Di Valentin C., Pacchioni G. (2015). Defect Calculations
in Semiconductors through a Dielectric-Dependent
Hybrid DFT Functional: The Case of Oxygen Vacancies in Metal Oxides. J. Chem. Phys..

[52] Kowalczyk D. A., Rogala M., Szałowski K., Kozłowski W., Lutsyk I., Piskorski M., Krukowski P., Dabrowski P., Belić D., Cichomski M., Kłusek Z., Kowalczyk P. J. (2021). Local Electronic
Structure of Stable
Monolayers of α-MoO3–x Grown on Graphite Substrate. 2D Mater..

[53] Mudgal S., Nayak M., Singh S., Komarala V. K. (2019). Study of
Anomalous
S-Shape in Current Density-Voltage Characteristics of Carrier Selective
Contact Molybdenum Oxide and Amorphous Silicon Based Heterojunction
Silicon Solar Cells. AIP Conf. Proc..

[54] Aghassi A., Fay C. D., Mozer A. (2018). Investigation of S-Shaped Current-Voltage
Characteristics in High-Performance Solution-Processed Small Molecule
Bulk Heterojunction Solar Cells. Org. Electron..

[55] Garcia-Sanchez, F. J. ; Romero, B. Equivalent Circuit Models for next Generation Photovoltaic Devices with S-Shaped i-v Curves. In 2019 8th International Symposium on Next Generation Electronics, ISNE; IEEE, 2019, pp. 1–4. DOI: 10.1109/ISNE.2019.8896544.

[56] Zuo L., Yao J., Li H., Chen H. (2014). Assessing the Origin of the S-Shaped
I-V Curve in Organic Solar Cells: An Improved Equivalent Circuit Model. Sol. Energy Mater. Sol. Cells.

[57] Scire, D. ; Bonadonna, M. ; Zhao, Y. ; Procel, P. ; Isabella, O. ; Zeman, M. ; MacAluso, R. ; Mosca, M. ; Crupi, I. Analysis of Transition Metal Oxides Based Heterojunction Solar Cells with S-Shaped J-V Curves. In 2020 AEIT International Annual Conference (AEIT); IEEE, 2020, pp. 1–6. DOI: 10.23919/AEIT50178.2020.9241142.

[58] Breitenstein O., Bauer J., Altermatt P. P., Ramspeck K. (2009). Influence of Defects
on Solar Cell Characteristics. Solid State Phenom..

