# Supplementary material for: Defect Density Analysis of WO x and MoO x Thin Films Grown by Pulsed Laser Deposition for Heterojunction Solar Cell Applications
Source: ACS Appl Energy Mater. 2025 Jun 23;8(13):9016–28. doi: 10.1021/acsaem.5c00629 (PMC12264978; doi:10.1021/acsaem.5c00629)
Supplement: Supplementary file 1 [file ae5c00629_si_001.pdf]

SUPPORTING INFORMATION

Defect density analysis of WO_x and MoO_x thin films grown by pulsed laser deposition for heterojunction solar cell applications

Daniele Scirè[1], Roberto Macaluso[1], Mauro Mosca[1], Maria Pia Casaletto[2], Olindo Isabella[3], Miro Zeman[3], and Isodiana Crupi[1]*

[1] Department of Engineering, University of Palermo, Viale delle Scienze, Ed. 9, Palermo, 90128, Italy

[2] Institute of Nanostructured Materials (ISMN), National Research Council (CNR), Via Ugo La Malfa 153, Palermo, 90146, Italy

[3] Photovoltaic Materials and Devices Group, Delft University of Technology, Mekelweg 4, Delft, 2628CD, the Netherlands

* Address correspondence to daniele.scire@unipa.it

Table S1. Pulsed laser deposition parameters and deposition conditions of the investigated samples.

PLD deposition conditionss	Value
Laser Wavelength	355 nm
Energy density (fluency)	1.2 J/cm^2
Repetition rate	20 Hz
Target composition	WO_3 99.9%, and MoO_3 99.9%
Substrate temperature	Room temperature, 200 °C, 400 °C
Oxygen background pressure	3×10^{-2}, 6×10^{-2}, 10×10^{-2} mbar

Table S2. XPS curve-fitting of W 4f and O 1s and surface relative chemical composition of the investigated samples. The two spin-orbit components (W $4f_{7/2}$ andW $4f_{5/2}$) of W 4f spectrum resulted energy split by Δ=2.14 eV. Elemental concentration is expressed as atomic percentage (at. %).

SAMPLE	BE (eV)		Concentration (at. %)	
PO_2,T_{dep}	W $4f_{7/2}$	W 4f $_{5/2}$	W	O
	O 1s		*O/W*	
3×10^{-2} mbar,	36.5	38.6	54.0	46.0
400 °C	530.6		*0.9*	

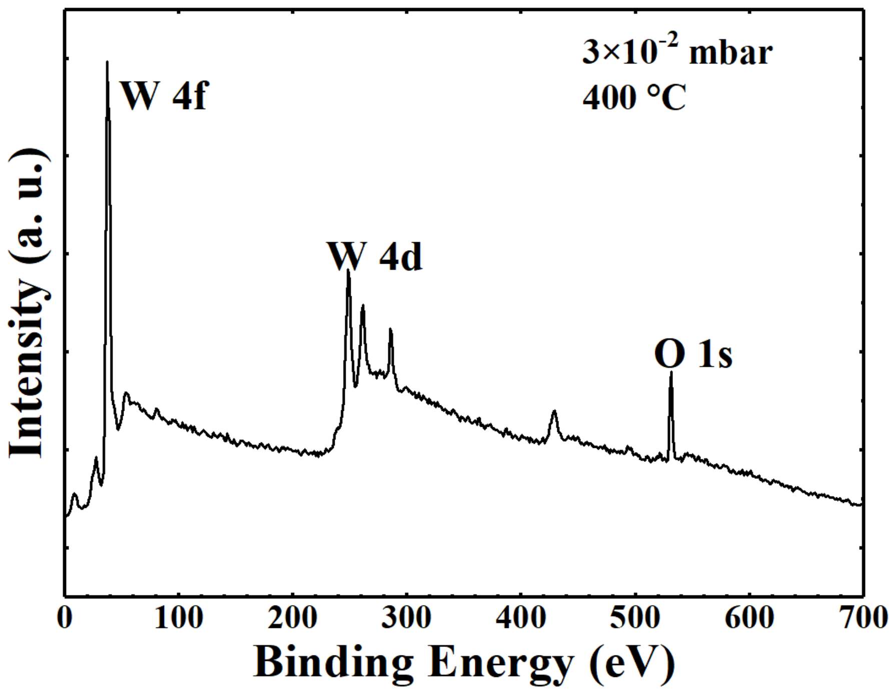

Figure S1. XPS wide scan spectrum of the WO_x sample deposited at T = 400 °C and $PO_2 = 3 \times 10^{-2}$ mbar.

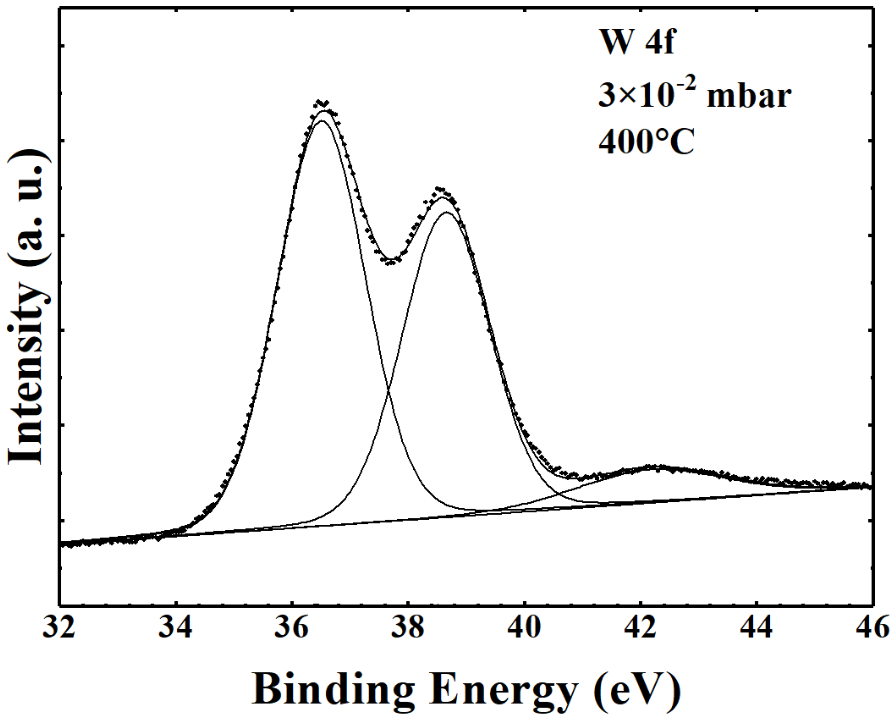

Figure S2. XPS curve fitting of the W 4f spectrum of the WO_x sample deposited at T = 400 °C and $PO_2 = 3 \times 10^{-2}$ mbar.

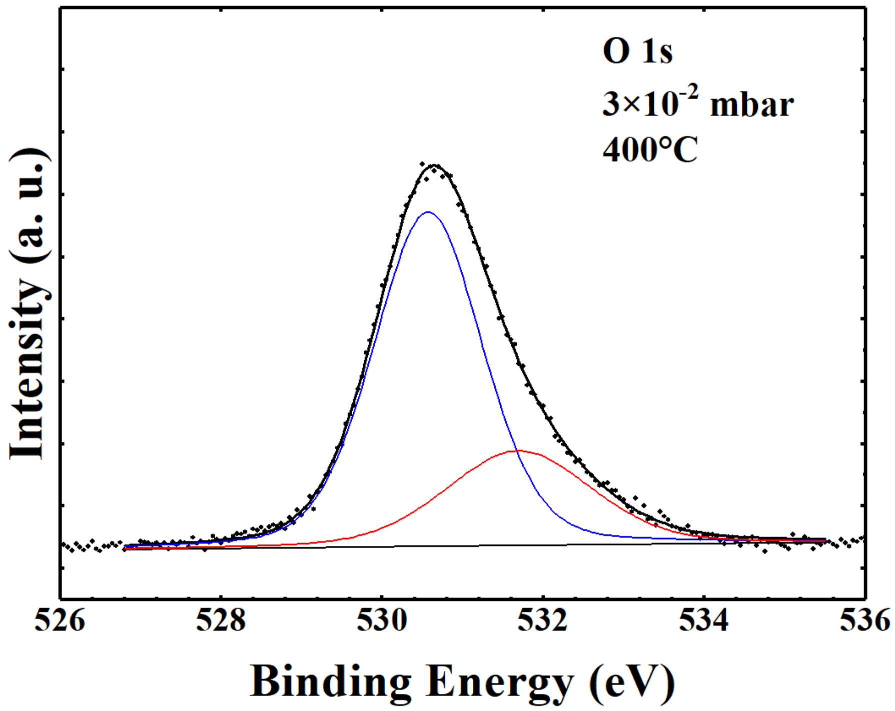

Figure S3. XPS curve fitting of the O 1s spectrum of the WO_x sample deposited at T = 400 °C and $PO_2 = 3 \times 10^{-2}$ mbar.

Table S3. XPS curve-fitting of Mo and O 1s and surface relative chemical composition of the investigated samples. The two spin-orbit components (Mo $3d_{5/2}$ and Mo $3d_{3/2}$) of Mo 3d spectrum were energy split by Δ~3.15 eV. Elemental concentration is expressed as atomic percentage (at. %).

**SAMPLE**	**BE (eV)**		**Concentration (at. %)**	
PO_2,T_{dep}	Mo $3d_{5/2}$	Mo $3d_{3/2}$	Mo	O
	O 1s		*O/Mo*	
3×10^{-2} mbar,	36.5	38.6	47.8	52.2
400 °C	530.6		*1.1*	

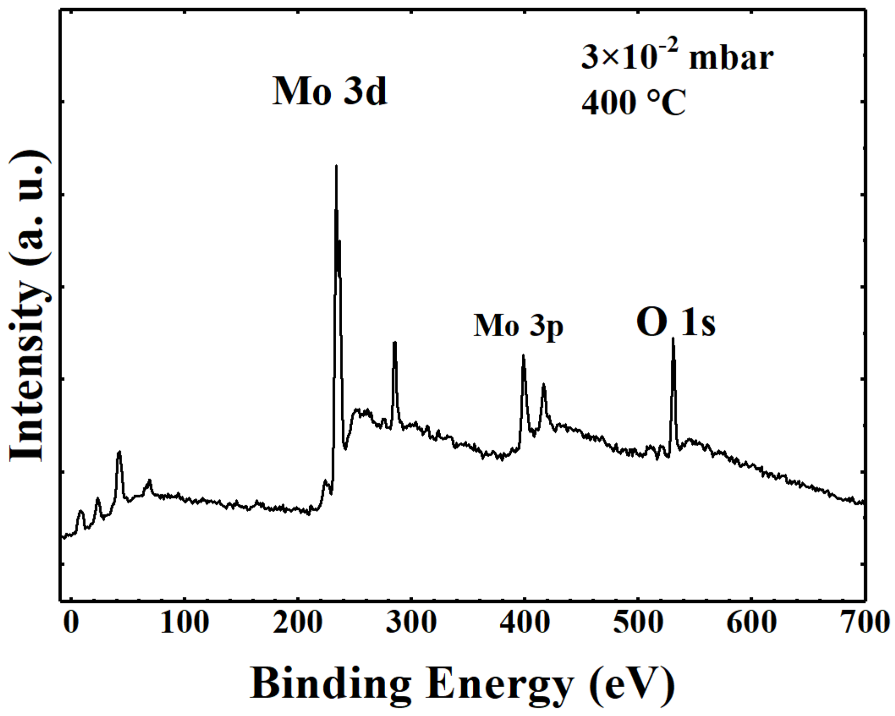

Figure S4. XPS wide scan spectra for the MoO_x sample deposited at T =400 °C and PO_2 = 3 × 10^{-2} mbar.

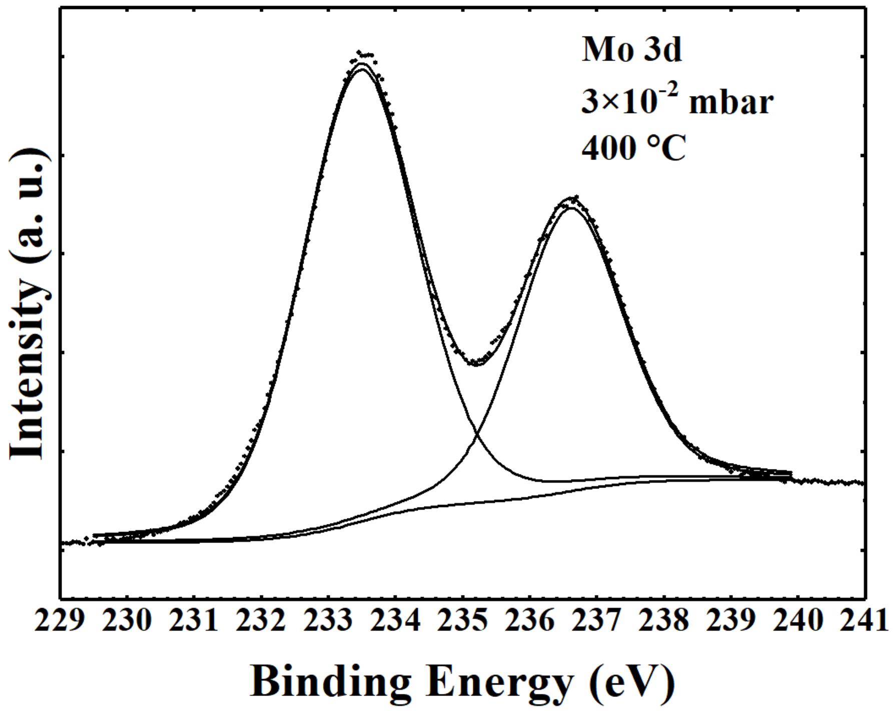

Figure S5. XPS curve fitting of the Mo 3d spectrum for the MoO_x sample deposited at 400 °C and PO_2 of 3 × 10^{-2} mbar.

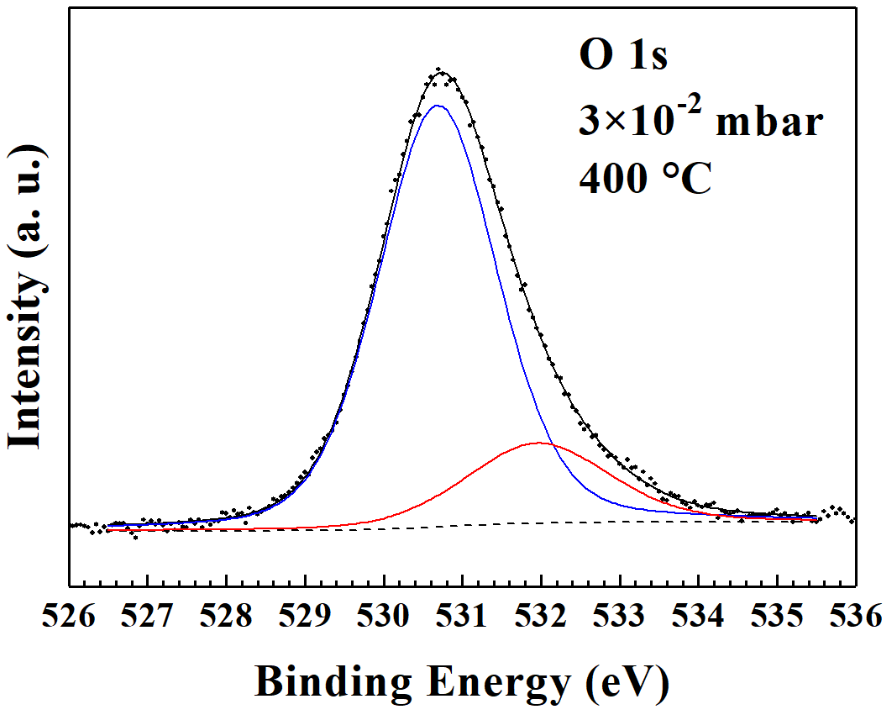

Figure S6. XPS curve fitting of the O 1s spectrum for the MoO_x sample deposited at T =400 °C and PO_2 = 3 × 10^{-2} mbar.

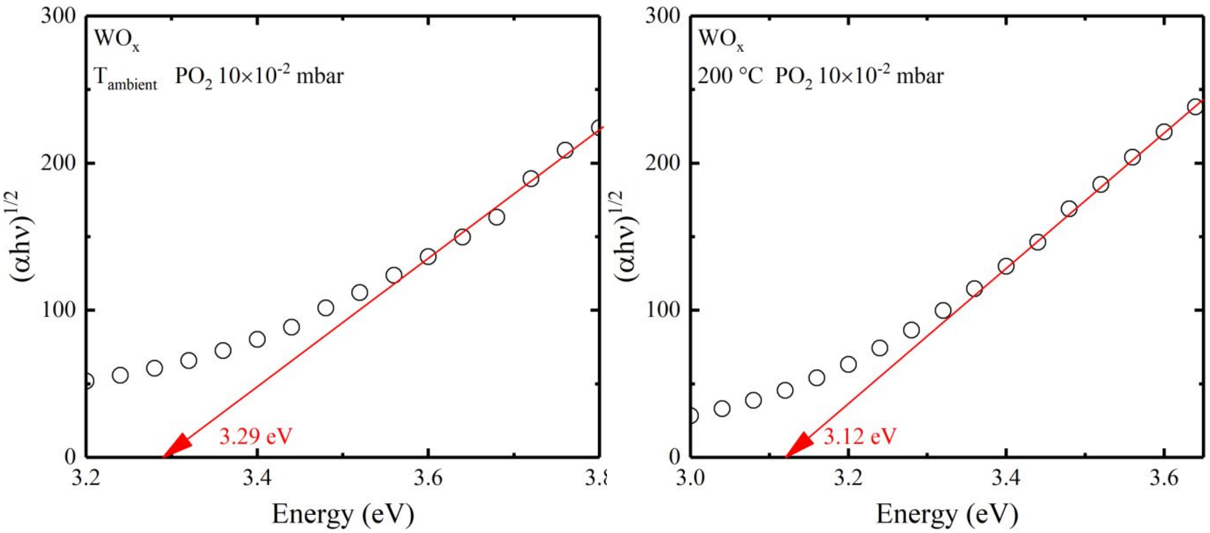

Figure S7. Tauc plot of $(\alpha h\nu)^{1/2}$ used for the estimation of the indirect optical bandgap in the tungsten oxide samples deposited at ambient temperature and at 200 °C with PO_2=10×10^{-2} mbar. The red arrows represent graphically the linear regression with the intercept on the x-axis highlighting the estimated gap value.

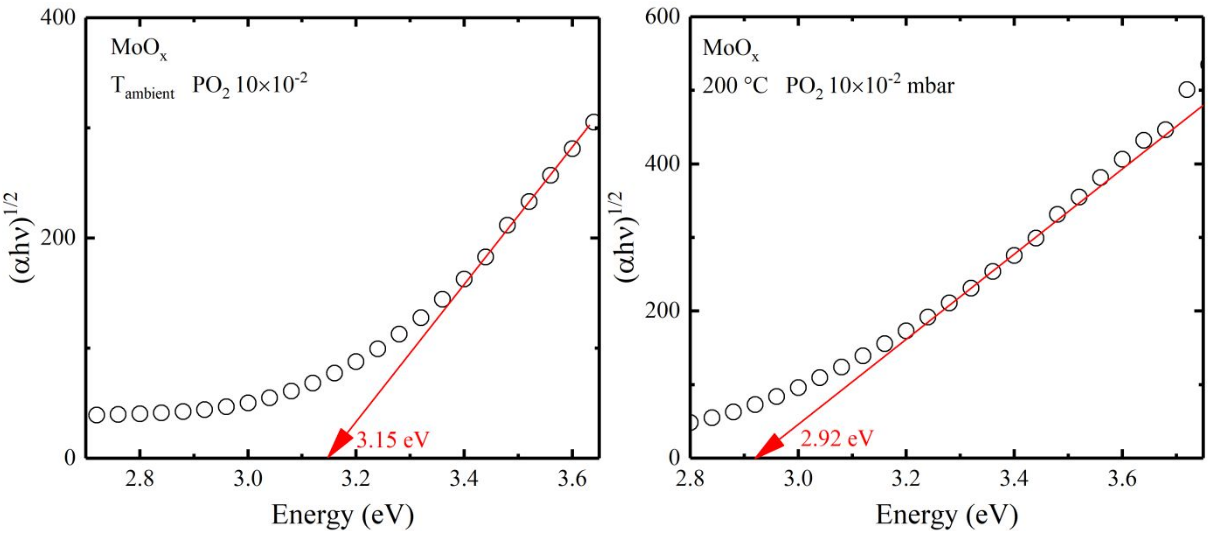

Figure S8. Tauc plot of $(\alpha h\nu)^{1/2}$ used for the estimation of the indirect optical bandgap in the molybdenum oxide samples deposited at ambient temperature and at 200 °C with PO_2=10×10^{-2} mbar. The red arrows represent graphically the linear regression with the intercept on the x-axis highlighting the estimated gap value.
